# Transcriptome profiling of posterior kidney of brown trout, *Salmo trutta*, during proliferative kidney disease

**DOI:** 10.1186/s13071-019-3823-y

**Published:** 2019-11-29

**Authors:** Arun Sudhagar, Reinhard Ertl, Gokhlesh Kumar, Mansour El-Matbouli

**Affiliations:** 1 Clinical Division of Fish Medicine, University of Veterinary Medicine, Vienna, Austria; 2 VetCore Facility for Research, University of Veterinary Medicine, Vienna, Austria; 3 Central Institute of Fisheries Education, Rohtak Centre, Rohtak, Haryana India

**Keywords:** Myxozoan, *Tetracapsuloides bryosalmonae*, Salmonids, RNA-seq, Gene expression

## Abstract

**Background:**

*Tetracapsuloides bryosalmonae* is a myxozoan parasite which causes economically important and emerging proliferative kidney disease (PKD) in salmonids. Brown trout, *Salmo trutta* is a native fish species of Europe, which acts as asymptomatic carriers for *T. bryosalmonae*. There is only limited information on the molecular mechanism involved in the kidney of brown trout during *T. bryosalmonae* development. We employed RNA sequencing (RNA-seq) to investigate the global transcriptome changes in the posterior kidney of brown trout during *T. bryosalmonae* development.

**Methods:**

Brown trout were exposed to the spores of *T. bryosalmonae* and posterior kidneys were collected from both exposed and unexposed control fish. cDNA libraries were prepared from the posterior kidney and sequenced. Bioinformatics analysis was performed using standard pipeline of quality control, reference mapping, differential expression analysis, gene ontology, and pathway analysis. Quantitative real time PCR was performed to validate the transcriptional regulation of differentially expressed genes, and their correlation with RNA-seq data was statistically analyzed.

**Results:**

Transcriptome analysis identified 1169 differentially expressed genes in the posterior kidney of brown trout, out of which 864 genes (74%) were upregulated and 305 genes (26%) were downregulated. The upregulated genes were associated with the regulation of immune system process, vesicle-mediated transport, leucocyte activation, and transport, whereas the downregulated genes were associated with endopeptidase regulatory activity, phosphatidylcholine biosynthetic process, connective tissue development, and collagen catabolic process.

**Conclusion:**

To our knowledge, this is the first RNA-seq based transcriptome study performed in the posterior kidney of brown trout during active *T. bryosalmonae* development. Most of the upregulated genes were associated with the immune system process, whereas the downregulated genes were associated with other metabolic functions. The findings of this study provide insights on the immune responses mounted by the brown trout on the developing parasite, and the host molecular machineries modulated by the parasite for its successful multiplication and release.

## Background

Proliferative kidney disease (PKD) is an emerging disease among salmonids caused by the extracellular myxozoan parasite *Tetracapsuloides bryosalmonae*. PKD is widely prevalent in Europe and North America [1]. PKD is of substantial importance, as it causes significant economic losses and ecological impacts in farmed and wild salmonids, respectively. Moreover, climate change driven rising water temperature can accelerate the *T. bryosalmonae* multiplication in fish which elevates the disease severity and associated mortalities, and also favours propagation of the parasite [2–4]. Scientific evidence points towards *T. bryosalmonae* as one of the major reasons for the protracted decline of endemic salmonids including brown trout (*Salmo trutta*) in the Alpine streams of Europe [5, 6]. In 2016 massive mortality of fishes, particularly mountain whitefish (*Prosopium williamsoni*) occurred due to *T. bryosalmonae* outbreak at the Yellowstone River, Montana, USA and a large section of the river was refrained from access to the public [7].

The life-cycle of *T. bryosalmonae* involves two hosts, an invertebrate bryozoan and a vertebrate salmonid fish [8, 9]. Infected bryozoans release parasite spores into the water, which enters the fish *via* gills. The entered parasite migrates *via* bloodstream to the kidney and undergoes extra-sporogonic proliferation and differentiation through sporogenesis [10, 11]. The sporogenesis of the parasite in the interstitial tissue of kidney results in the proliferation of leucocytes and granulomatous cellular response, which leads to the swelling of kidney [12–14]. The mature parasitic spores are released by infected fish, mainly brown trout and brook trout, *via* urine into the aquatic environment, and readily infect bryozoans [9].

Apart from excretory function, teleost kidney serves as complex multifunctional immune organ. Teleosts lack bone marrow and the kidney is analogous to mammalian bone marrow for immune function [15]. Anterior kidney of teleosts performs haematopoiesis, production and maturation of B lymphocytes. The matured B lymphocytes then migrate to either spleen or posterior kidney for activation [16]. Interestingly, *T. bryosalmonae* can develop and multiply in the kidney of salmonids, which is an active site of immune response.

Previous studies have demonstrated that the European strain of *T. bryosalmonae* has co-evolved with the endemic brown trout host in which the parasite can complete the life-cycle, whereas the rainbow trout (*Oncorhynchus mykiss*) cannot release the mature spores and remains as a dead end fish host [17]. In addition, the parasite can establish long-term persistence in brown trout and shed contagious spores even after five years post-infection [18]. Rainbow trout has been extensively studied in response to PKD and the disease pathogenesis is defined by a profound dysregulation of B cell subsets and cytokines of T-helper cells, decrease of myeloid cells and increase of lymphocytes, and overexpression of suppressors of cytokine signaling (SOCS) genes [4, 19–25]. However, only few gene expression studies have been investigated in the kidney of PKD-affected brown trout [24, 26]. Although these studies are important, global transcriptome analysis of the posterior kidney of brown trout during PKD is still needed to visualize a broader picture during host-parasite interaction. Furthermore, there are still many open questions about the molecular factors of brown trout, influenced for successful proliferation, chronic subclinical persistence and release of *T. bryosalmonae*. RNA sequencing (RNA-seq)-based transcriptome analysis can provide insights and serve as a valuable tool to understand the host-parasite interaction [27].

Here, we employed transcriptome analysis in search of meaningful biological insights of transcripts during *T. bryosalmonae* proliferation in the posterior kidney of brown trout. Furthermore, we displayed the global kidney transcriptome responses by defining the most relevant gene ontology terms and pathways involved in PKD pathogenesis.

## Methods

### Brown trout maintenance

Specific pathogen-free (SPF) brown trout (mean length 12 ± 2 cm) were procured from a certified Austrian hatchery and acclimatized for 4 weeks. Fish (*n* = 10) were tested randomly for the presence of bacterial, viral and parasitic infection including *T. bryosalmonae* by our routine diagnostic procedures including PCR. All the tested fish were observed to be disease-free. Prior to the experiment, fish were acclimatized in 1000-l tanks with continuous flow through freshwater system with water temperature 15 ± 1 °C and fed *ad libitum* with commercial trout feed.

### Brown trout experiment

SPF brown trout (*n* = 69) were divided equally to three tanks (23 fish per tank). Fish from each tank were exposed to parasite spores released from the parasite sacs (*n* = 150), according to Kumar et al. [28]. At the same time, 69 SPF brown trout were maintained separately as unexposed control. The fish were maintained in 100-l aquarium with continuous flow through freshwater system at 15 ± 1 °C with sufficient feeding. Moribund fish were immediately removed from the parasite-exposed tanks and were euthanized. For each sampling, the fish (*n* = 9) from unexposed and exposed groups were euthanized using buffered MS-222 anesthetic (Sigma-Aldrich, Steinheim, Germany). Blood and different organs including posterior kidney were sampled at 2, 4, 6, 8, 10, 12 and 17 weeks post-exposure (wpe). The organs of each fish were divided into two portions, one fixed in 10% neutral-buffered formalin for histological investigation, and the second portion fixed in RNA*later* (Sigma-Aldrich) or used to purify parasites for molecular studies.

### RNA extraction, library preparation and sequencing

**Fig. 1:**
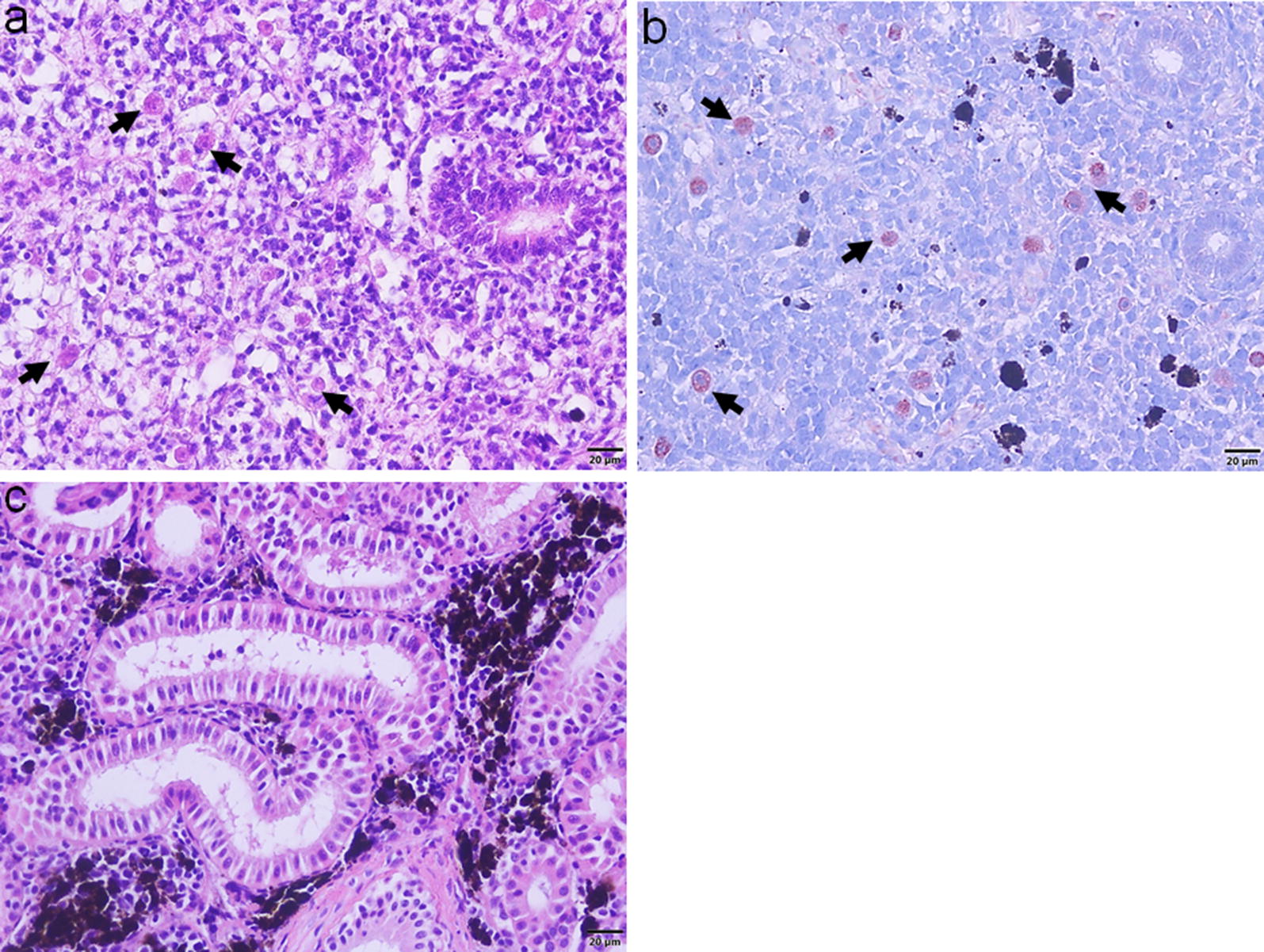
Histological sections of brown trout posterior kidney. **a** *Tetracapsuloides bryosalmonae-*exposed posterior kidney section shows *T. bryosalmonae* (arrows) proliferation, pronounced tubular degeneration and reduction of melanomacrophages at 12 weeks post-exposure (H&E staining). **b** Immunohistostaining shows interstitial extrasporogonic stages of *T. bryosalmonae* (arrows) in the posterior kidney. **c** No parasite or any renal changes were observed in the unexposed posterior control kidney (H&E staining). *Scale-bars*: 20 µm

The optimal time point for the RNA-seq was determined by the presence of numerous interstitial proliferating *T. bryosalmonae* in the posterior kidney detected using histology and immunohistochemistry [28]. The parasite-exposed brown trout kidney samples collected at 12 wpe had pronounced necrotic changes, degenerated renal tubules and reduction of melanomacrophages along with numerous interstitial pre-sporogenic stages of *T. bryosalmonae* (Fig. 1a, b). No parasite or renal changes were observed in the unexposed control kidney samples (Fig. 1c).

Twelve cDNA libraries (for 6 exposed and 6 unexposed control fish) were generated and sequenced. Briefly, total RNA was extracted from the posterior kidney samples of exposed (*n* = 6) and unexposed control (*n* = 6) brown trout using RNeasy Mini Kit (Qiagen, Hilden, Germany) with an on-column DNase digestion step. The purity and integrity of the extracted RNA were accessed with a 4200 TapeStation (Agilent, Santa Clara, CA, USA) using the RNA ScreenTape assay. All the samples had RIN values above 7.0 and were used for cDNA library preparation. Briefly, 500 ng total RNA per sample were used for library preparation with the TruSeq RNA Library Prep Kit v2 (Illumina, San Diego, CA, USA) according to the manufacturer’s protocol. Library quality control was done with the D1000 ScreenTape assay (Agilent, Santa Clara, CA, USA) on the 4200 TapeStation. All the 12 cDNA libraries were sequenced (100-bp single reads) on two lanes of an Illumina HiSeq 2500 platform.

### Mapping and differential gene expression analysis

The sequence data were analyzed using CLC Genomics Workbench 12 (Qiagen, Aarhus, Denmark). The raw sequence reads were subjected to quality (limit = 0.05 and maximum 2 ambiguous nucleotides allowed) and adapter trimming. Reads shorter than 25 nucleotides were discarded. Currently, no genome data are available for brown trout and hence, *de novo* assembled brown trout transcriptome (GenBank: GFIS00000000) taken from the NCBI TSA database were used as a reference [29]. The trimmed reads were mapped against the transcriptome reference using the default mapping parameters of the CLC Genomics RNA-seq tool. Additionally, for comparison, mapping was done using the genomes of the closely related rainbow trout (GenBank: GCA_002163495.1) and Atlantic salmon (*Salmo salar*) (GenBank: GCA_000233375.4) as reference. However, the mapping percentage was higher with brown trout transcriptome (90%) compared to rainbow trout genome (76%) and Atlantic salmon genome (84%) (Table 1). Therefore, brown trout transcriptome was considered a better reference and further used for differential expression analysis of posterior kidney. Read counts of parasite-exposed kidney samples were compared with unexposed control kidney samples using the Empirical analysis of DGE tool which implements the Exact Test for two-group comparisons in the CLC Genomics Workbench [30]. A total count filter cut-off value of 5.0 was set to exclude features with low read counts across all samples. All features showing a false discovery rate, adjusted *P*-value < 0.01 and fold change ≥ |2| were considered as differentially expressed. Moreover, global Pearsonʼs correlation was analysed between samples for log_2_ (TPM + 1) normalized RNA-seq values using *ggcorrplot* package [31]. The differentially expressed contigs including non-annotated transcripts were re-annotated using NCBI BLASTX and BLASTN tools with a cut-off threshold of E-value 1.0E−3 in the NCBI non-redudant database using Blast2GO [32].

**Table 1:** Description of transcriptome data of *Tetracapsuloides bryosalmonae*-exposed and unexposed control posterior kidney of brown trout

Samples	Total no. of reads	No. of trimmed reads	Mapping with rainbow trout reference genome				Mapping with Atlantic salmon reference genome				Mapping with brown trout reference transcriptome			
			No. of mapped reads	% of mapped reads	No. of unmapped reads	% unmapped reads	No. of mapped reads	% of mapped reads	No. of unmapped reads	% unmapped reads	No. of mapped reads	% of mapped reads	No. of unmapped reads	% unmapped reads
Control 1	37,453,251	37,432,308	28,587,181	76.37	8,845,127	23.63	31,555,799	84.3	5,876,509	15.7	33,957,176	90.72	3,475,132	9.28
Control 2	36,014,039	36,000,623	27,596,056	76.65	8,404,567	23.35	30,167,749	83.8	5,832,874	16.2	32,695,497	90.82	3,305,126	9.18
Control 3	33,756,491	33,742,632	25,599,517	75.87	8,143,115	24.13	28,588,179	84.72	5,154,453	15.28	30,631,472	90.78	3,111,160	9.22
Control 4	28,906,028	28,893,551	22,082,023	76.43	6,811,528	23.57	24,154,386	83.6	4,739,165	16.4	26,126,636	90.42	2,766,915	9.58
Control 5	35,936,239	35,917,599	27,065,039	75.35	8,852,560	24.65	30,203,792	84.09	5,713,807	15.91	32,698,686	91.04	3,218,913	8.96
Control 6	35,784,560	35,769,721	27,665,605	77.34	8,104,116	22.66	30,149,740	84.29	5,619,981	15.71	32,632,620	91.23	3,137,101	8.77
Exposed 1	36,037,693	36,021,748	26,712,983	74.16	9,308,765	25.84	30,604,349	84.96	5,417,399	15.04	32,234,190	89.49	3,787,558	10.51
Exposed 2	36,585,106	36,570,194	27,967,577	76.48	8,602,617	23.52	30,928,270	84.57	5,641,924	15.43	33,142,773	90.63	3,427,421	9.37
Exposed 3	34,790,815	34,777,577	26,339,581	75.74	8,437,996	24.26	29,540,271	84.94	5,237,306	15.06	31,484,762	90.53	3,292,815	9.47
Exposed 4	34,863,462	34,848,992	26,601,431	76.33	8,247,561	23.67	29,178,501	83.73	5,670,491	16.27	31,894,763	91.52	2,954,229	8.48
Exposed 5	35,989,820	35,973,511	27,781,132	77.23	8,192,379	22.77	30,067,422	83.58	5,906,089	16.42	32,541,899	90.46	3,431,612	9.54
Exposed 6	35,705,332	35,687,549	27,935,659	78.28	7,751,890	21.72	29,067,347	81.45	6,620,202	18.55	32,373,706	90.71	3,313,843	9.29

*Notes*: Filtered clean reads were mapped against the available genomes of rainbow trout and Atlantic salmon, and brown trout reference transcriptome

### Gene ontology, enrichment and pathway analysis

Differentially expressed genes (DEGs) were subjected to gene ontology (GO) and enrichment analysis for biological process, molecular function, and cellular components. This was performed in the ClueGO version 2.5.4 plugin [33] of the Cytoscape version 3.7.1 software platform [34]. Due to the non-availability of GO data for brown trout, the analysis was performed based on human GO data. Therefore, all the brown trout gene identifiers were converted to HUGO nomenclature and provided as input in ClueGO [35]. The analysis was done for up- and downregulated genes, separately. A minimum of three genes were used as the cut-off to find the GO term and two-sided hypergeometric statistical testing corrected with the Bonferoni step-down method (*P* < 0.05) and a kappa score of four was used as the cut-off. Furthermore, the DEGs were analyzed in Kyoto Encyclopedia of Genes and Genomes (KEGG) pathway database using online KEGG mapper tool under the reference pathway, KO [36].

### Validation of genes by quantitative real time PCR

Twelve DEGs were selected to validate the expression profile of RNA-seq such as complement C1q like-2 (C1QL2), calcium-binding protein S100-A1 (S100A1), Mucin-7 (MUC7), C-X-C chemokine receptor type 1-like (CXCR1), C-C chemokine receptor type 5-like (CCR5), H-2 class II histocompatibility antigen gamma chain-like (CD74), cathepsin-B (CTSB), apelin receptor A-like (APLNR), PEX5-related protein-like (PEX5L), matrix metallopeptidase 28 (MMP28), solute carrier family 16 member 4 (SLC16A4) and tomoregulin-1-like (TMEFF1). Gene-specific primers were designed according to sequence data of the kidney transcriptome using NCBI Primer-BLAST online tool. One µg of total RNA was used for the synthesize of cDNA using iScript cDNA Synthesis Kit (Bio-Rad, Hercules, USA). The cDNA samples of exposed and unexposed control posterior kidneys (*n* = 6) were subjected to quantitative real time PCR (qRT-PCR) with two technical replicates using the optimized gene primers (Additional file 1). qRT-PCR was performed in a final volume of 20 μl, which contained 4 μl of 1:10-fold diluted cDNA, 0.5 μM of each primer, 1× SsoAdvanced™ Universal SYBR Green Supermix (Bio-Rad) and DEPC-treated sterile distilled water. After 5 min of cDNA denaturation at 95 °C, 37 cycles were performed at 95 °C for 30 s, 57 °C for 30 s and 72 °C for 30 s in a CFX96 Touch Real-Time PCR detection system (Bio-Rad, München, Germany). At the end of all gene expression cycling protocols, melting curve analysis was performed to validate amplification specificity under the following conditions: 57 °C for 30 s to 95 °C with an increment of 0.5 °C for 10 s. Elongation factor alpha [28] was used as a reference gene to normalize the test samples. The 2^−ΔΔCt^ method was calculated to determine the relative gene expression presented as the fold increase or decrease of the exposed group relative to the unexposed control group (mean expression level adjusted to 1). The statistical difference between groups was determined using the two-tailed unpaired Student’s t-test with Welch’s correction. Linear regression analysis was performed on corresponding log_2_ fold change values of RNA-seq and qRT-PCR to evaluate the relationship between them. For all statistical tests, *P*-value of < 0.05 was regarded as significant and the data were analyzed in R statistical software version 3.5.1 [37].

## Results

### Mapping of sequence reads and analysis of differentially expressed genes

**Fig. 2:**
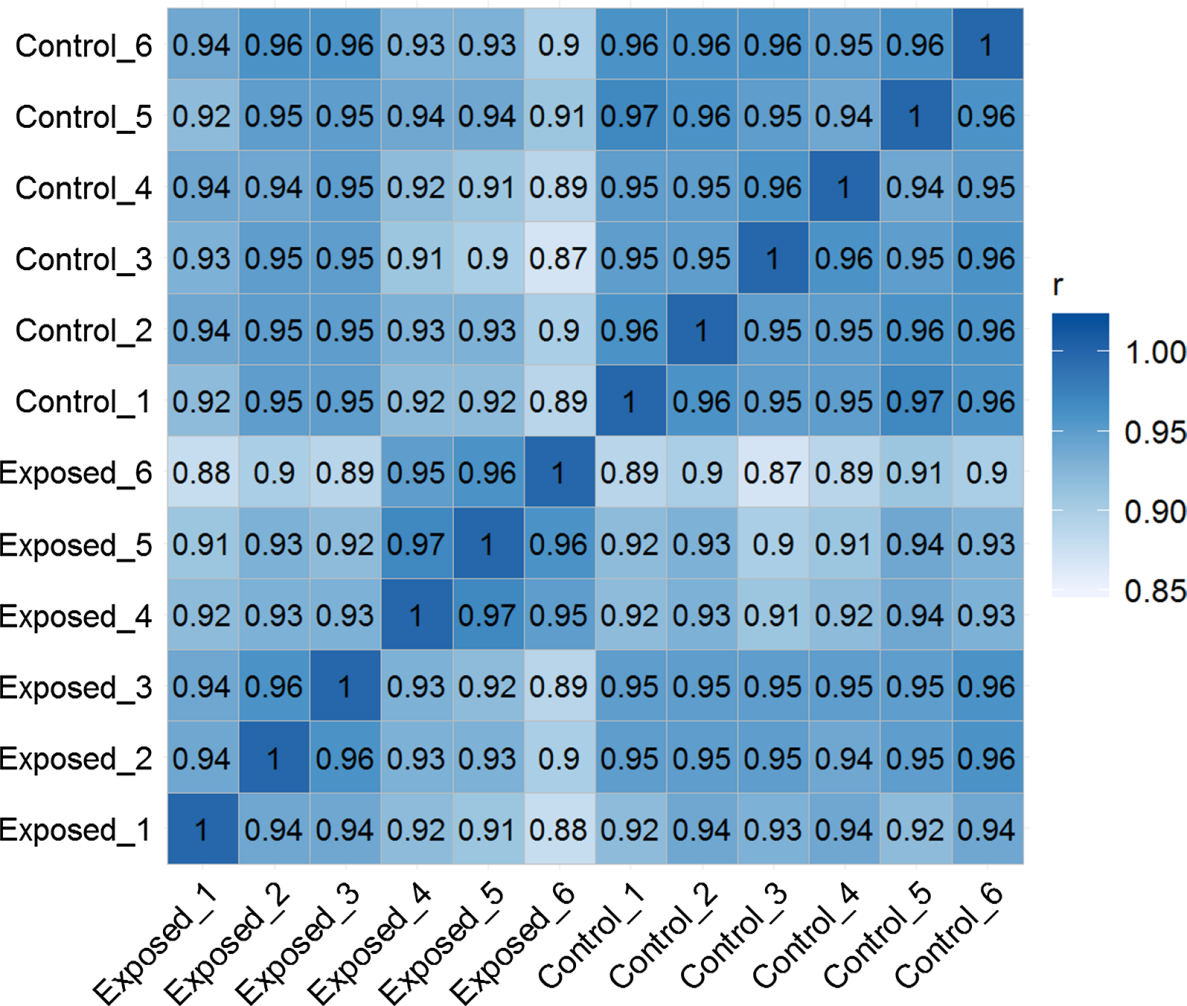
Global correlation matrix of gene expression levels between samples. Heatmap showing Pearsonʼs correlation coefficient (*r*) for log_2_ (TPM + 1) normalized RNA-seq values across samples, indicating positive correlation between biological replicates

**Fig. 3:**
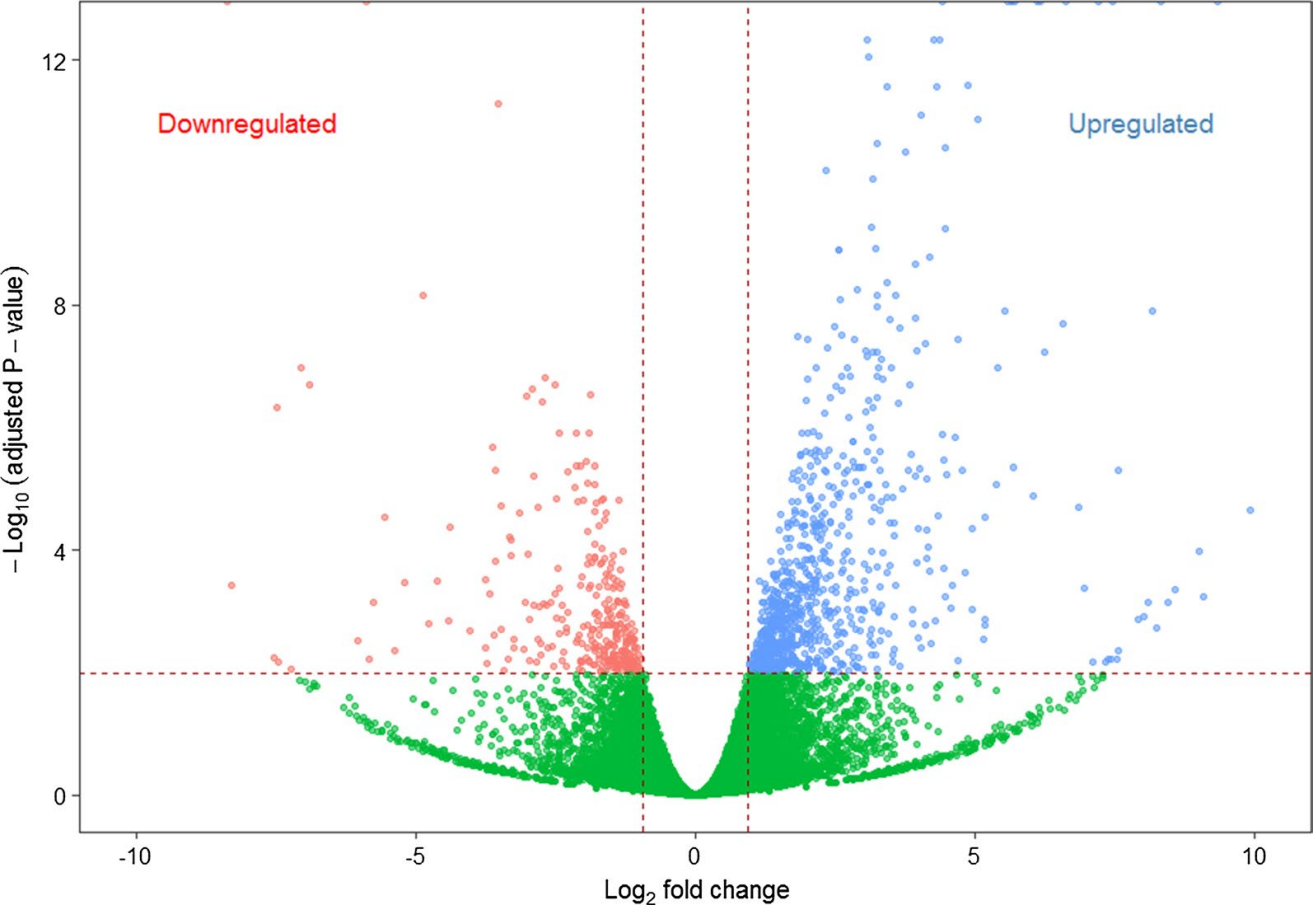
Volcano plot of differently expressed genes of posterior kidney. x-axis represents log_2_ transformed fold change and the y-axis indicates − log_10_ transformed adjusted significance. Each dot indicates an individual gene that is significantly upregulated genes (blue), downregulated genes (red) and non-significantly regulated genes (green). The horizontal line represents adjusted *P*-value < 0.01 cut-off and the vertical lines represent the absolute value of fold change greater than or equal to two

**Fig. 4:**
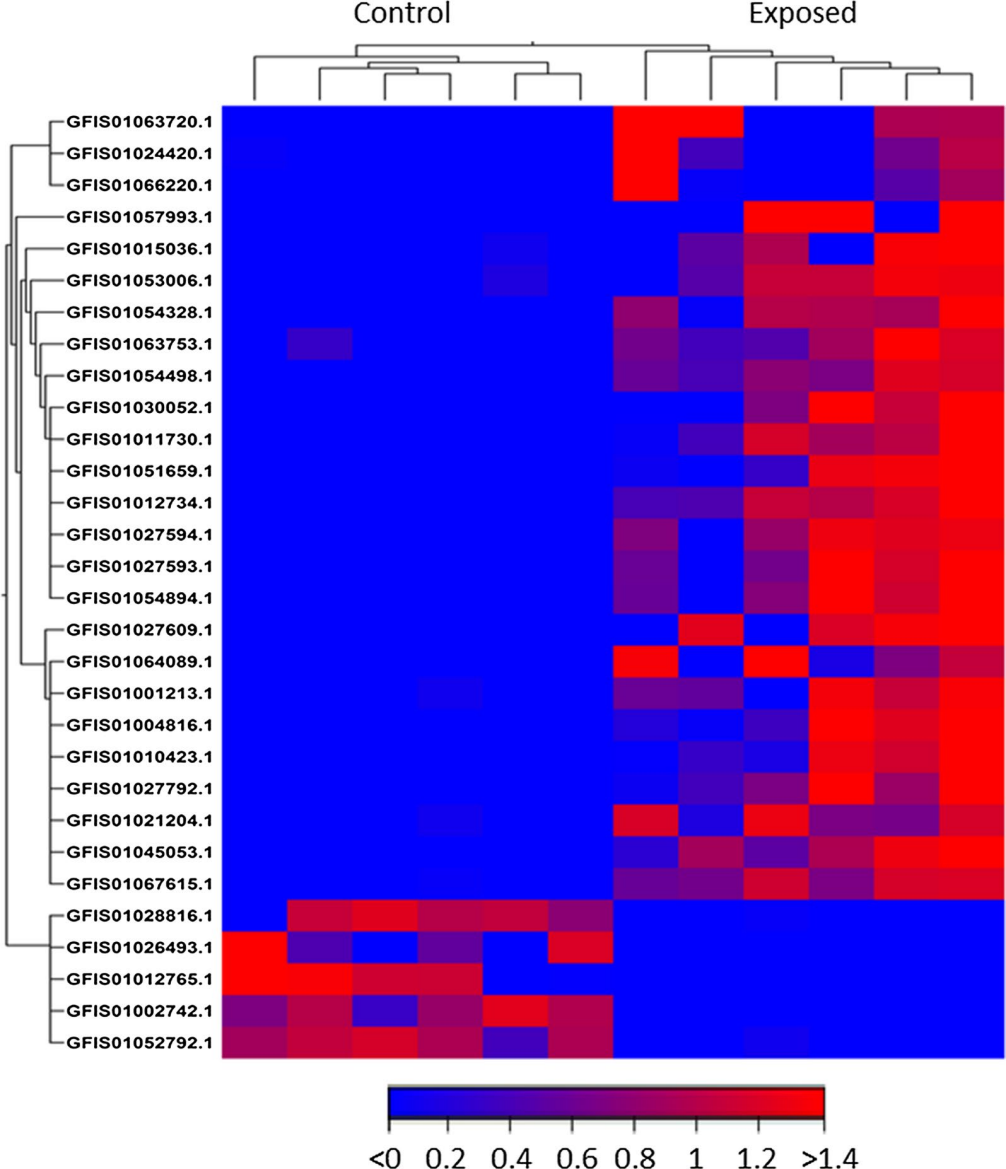
Heatmap visualization and hierarchical clustering of selected 30 differentially expressed genes. The heatmap displays selected 30 differentially expressed genes between *Tetracapsuloides bryosalmonae-*exposed and unexposed control brown trout posterior kidney selected based on adjusted *P*-value, sample and feature. Hierarchical clustering was performed using the single linkage method based on Euclidean distance matrix. Each column represents a posterior kidney sample and each row represents a gene

A total of 421.6 million clean single-end reads with a length of 100 bases were obtained by sequencing all 12 libraries. Approximately, 28.89 to 37.43 million single-end reads were obtained from each library (Table 1). The clean reads mapped to 74,449 out of 75,257 contigs (98.2%) in the reference transcriptome and none of the reads mapped to 808 contigs in the reference transcriptome. Furthermore, global correlation analysis of expression levels between samples showed positive correlation between the biological replicates (Fig. 2). Comparison between exposed and unexposed control groups revealed 1169 DEGs (fold change ≥ |2|, adjusted *P*-value < 0.01), out of 75,257 contigs present on the reference brown trout transcriptome assembly. This accounts for 1.55% of the total contigs available in the reference brown trout transcriptome. DEGs were visualized in a heatmap and a volcano plot, which show that the number of upregulated DEGs were higher than the downregulated DEGs (Figs. 3, 4). Out of 1169 DEGs, 864 genes (74%) were upregulated and 305 genes (26%) were downregulated (Additional file 2). DEGs related to immune system and disease process were identified using GO and KEGG pathway analysis.

### Functional annotation of differentially expressed up- and downregulated genes

Out of 1169 DEGs, gene identifiers could be assigned for 1075 transcripts (91.95%). Majority of them were associated with the biological processes such as regulation of immune system process (58.54%), vesicle-mediated transport (9.76%), leucocyte activation (9.76%), cellular response to unfolded proteins (5.69%), and transport (4.88%). GO terms related to immune system were most enriched among the upregulated genes. The upregulated genes were part of cellular components such as vesicle (63.16%), side membrane (15.79%), endomembrane system (13.16%), cytosol (2.63%), cytoplasmic parts (2.63%), and actin cytoskeleton (2.63%). The upregulated genes were involved in molecular functions such as cytokine receptor activity (20%), binding identical proteins (20%), peptides (20%), enzymes (20%), and cytoskeleton proteins (20%) (Additional files 3, 4).

The downregulated genes were associated with the biological processes such as endopeptidase regulatory activity (20%), phosphatidylcholine biosynthetic process (20%), connective tissue development (10%), protein polymerization (10%), hormone metabolic process (10%), collagen catabolic process (10%), monocarboxylic acid transport (10%), and female pregnancy (10%). Moreover, within cellular components category the downregulated genes were associated with melanosome (38.46 %), cluster of actin-based cell projections (15.38%), cell-cell contact zone (7.69%), sarcolemma (7.69%), endosome lumen (7.69%), sarcoplasm (7.69%), spectrin-associated cytoskeleton (7.69%), and lipid droplet (7.69%). In addition, the downregulated genes have molecular functions such as endopeptidase regulator activity (55.56%), proteoglycan (22.22%), spectrin binding (11.11%), and symporter activity (11.11%) (Additional files 3, 5).

### KEGG pathway

The KEGG pathway assigned 571 out of 1169 DEGs (48.8%) to 282 pathways in the KEGG database (Additional file 6). Out of these 282 pathways, 21 were categorized under immune system pathways (Table 2). Among the immune-related pathways, the most DEGs were assigned to chemokine signaling pathway followed by NOD-like receptor signaling, natural killer cell mediated cytotoxicity, toll-like receptor signaling, and C-type lectin receptor signaling pathways. The list of immune genes assigned in representative immune related pathways are shown in Table 3. In addition to immune-related pathways the DEGs were also assigned to metabolic (35 genes), cytokine-cytokine receptor interaction (27 genes), JAK-STAT signaling (13 genes), endocytosis (11 genes), necroptosis (10 genes), and apoptosis (9 genes) pathways.

**Table 2:** Distribution of the differentially expressed genes in immune related pathways categorized from the KEGG pathway database

Sl No	Pathway ID	Pathway	No. of genes
1	ko04062	Chemokine signaling	12
2	ko04621	NOD-like receptor signaling	10
3	ko04650	Natural killer cell mediated cytotoxicity	9
4	ko04620	Toll-like receptor signaling	9
5	ko04625	C-type lectin receptor signaling	9
6	ko04659	Th17 cell differentiation	8
7	ko04640	Hematopoietic cell lineage	8
8	ko04662	B cell receptor signaling	7
9	ko04670	Leukocyte transendothelial migration	6
10	ko04658	Th1 and Th2 cell differentiation	6
11	ko04610	Complement and coagulation cascades	6
12	ko04672	Intestinal immune network for IgA production	5
13	ko04660	T cell receptor signaling	5
14	ko04666	Fc gamma R-mediated phagocytosis	5
15	ko04622	RIG-I-like receptor signaling	4
16	ko04657	IL-17 signaling	4
17	ko04611	Platelet activation	4
18	ko04612	Antigen processing and presentation	3
19	ko04623	Cytosolic DNA-sensing	3
20	ko04624	Toll and Imd signaling	2
21	ko04664	Fc epsilon RI signaling	2

*Notes*: The pathway analysis mapped 571 out of 1169 DEGs (48.8%) to 282 pathways, including 21 immune system pathways in the KEGG database

**Table 3:** List of the differentially expressed genes mapped to representative immune-related pathways by KEGG pathway analysis

Sl no	Gene code	Description	Fold change	Regulation
Chemokine signaling				
1	ADCY9	Adenylate cyclase type 9	3.59	Up
2	CCL11	C-C motif chemokine 11/eotaxin	9.56	Up
3	CCL4	C-C motif chemokine 4	13.42	Up
4	CCR5	C-C chemokine receptor type 5-like	4.31	Up
5	CXCL12	Stromal cell-derived factor 1 precursor	3.0	Up
6	CXCL13	C-X-C motif chemokine 13-like	4.99	Up
7	CXCR1	C-X-C chemokine receptor type 1-like	4.47	Up
8	CXCR3	C-X-C chemokine receptor type 3-like	− 2.49	Down
9	CXCR5	C-X-C chemokine receptor type 5-like	2.64	Up
10	FOXO3	Forkhead box O3	4.36	Up
11	GNG13	Guanine nucleotide-binding protein G(I)/G(S)/G(O) subunit gamma-13	3.53	Up
12	PXN	Paxillin-like	3.11	Up
NOD-like receptor signaling				
1	ATG16L1	Autophagy related 16-like 1	2.88	Up
2	ATG5	Autophagy related 5 homolog	4.89	Up
3	CASR	Extracellular calcium-sensing receptor-like protein	3.26	Up
4	CTSB	Cathepsin B	− 3.01	Down
5	IFNAR1	Interferon alpha/beta receptor 1a-like	2.34	Up
6	IFNAR2	Interferon alpha/beta receptor 2	2.72	Up
7	JUN	Transcription factor AP-1-like	3.75	Up
8	NLRP3	NACHT, LRR and PYD domains-containing protein 3	− 2.37	Down
9	PYCARD	Apoptosis-associated speck-like protein containing a CARD	2.53	Up
10	RIPK3	Receptor-interacting serine/threonine-protein kinase 3	2.70	Up
Natural killer cell mediated cytotoxicity				
1	BID	BH3-interacting domain death agonist-like	2.77	Up
2	ICAM1	Intercellular adhesion molecule 1-like isoform X2	− 7.45	Down
3	IFNAR1	Interferon alpha/beta receptor 1a-like	2.34	Up
4	IFNAR2	Interferon alpha/beta receptor 2	2.72	Up
5	IFNGR1	Interferon gamma receptor 1a	2.33	Up
6	IGH	Immunoglobulin heavy chain	147.29	Up
7	ITGB2	Integrin beta-2-like	3.0	Up
8	PRF1	Perforin-1-like	− 3.18	Down
9	PTPN11	Tyrosine-protein phosphatase non-receptor type 11	2.81	Up
Th17 cell differentiation				
1	CD3E	CD3epsilon	5.68	Up
2	IFNGR1	Interferon gamma receptor 1a	2.33	Up
3	IL21R	Interleukin-21 receptor-like	2.69	Up
4	IL6ST	Interleukin-6 receptor subunit beta-like	3.28	Up
5	IRF4	Interferon regulatory factor 4	7.28	Up
6	JUN	Transcription factor AP-1-like	3.75	Up
7	NFKBIE	Nuclear factor of kappa light polypeptide gene enhancer in B-cells inhibitor, epsilon	9.05	Up
8	TGFBR1	TGF-beta receptor type-1	− 2.14	Down
Hematopoietic cell lineage				
1	CD2	T-cell surface antigen CD2	2.89	Up
2	CD22	B-cell receptor CD22-like	3.10	Up
3	CD34	Hematopoietic progenitor cell antigen CD34-like	− 3.13	Down
4	CD3E	CD3epsilon	5.68	Up
5	CR2	Complement receptor type 2-like	3.25	Up
6	IGH	Immunoglobulin heavy chain	147.29	Up
7	MME	Neprilysin-like	− 4.06	Down
8	TFRC	Transferrin receptor protein 1-like	4.97	Up
B cell receptor signaling				
1	BLNK	B-cell linker protein-like isoform X1	3.43	Up
2	CD22	B-cell receptor CD22-like	3.10	Up
3	CD79A	B-cell antigen receptor complex-associated protein alpha chain-like	2.76	Up
4	CR2	Complement receptor type 2-like	3.25	Up
5	IGH	Immunoglobulin heavy chain	147.29	Up
6	JUN	Transcription factor AP-1-like	3.75	Up
7	NFKBIE	Nuclear factor of kappa light polypeptide gene enhancer in B-cells inhibitor, epsilon	9.05	Up
Th1 and Th2 cell differentiation				
1	CD3E	CD3 epsilon	5.68	Up
2	IFNGR1	Interferon gamma receptor 1a	2.33	Up
3	IL12B	Interleukin-12 subunit beta-like	2.31	Up
4	IL12RB2	Interleukin-12 receptor subunit beta-2-like	3.50	Up
5	JUN	Transcription factor AP-1-like	3.75	Up
6	NFKBIE	Nuclear factor of kappa light polypeptide gene enhancer in B-cells inhibitor, epsilon	9.05	Up
Complement and coagulation cascades				
1	A2M	Alpha-2-macroglobulin-like	− 7.82	Down
2	C8G	Complement C8 gamma chain	− 5.78	Down
3	CR2	Complement receptor type 2-like	3.25	Up
4	F2RL2	Proteinase-activated receptor 3-like (coagulation factor II (thrombin) receptor)	5.81	Up
5	ITGB2	Integrin beta-2-like	3.0	Up
6	PLAUR	Urokinase plasminogen activator surface receptor-like	− 3.17	Down

*Notes*: Chemokine signaling pathway has the predominant share of differentially expressed genes among the immune related pathways. Interestingly, most of the immune genes were upregulated in the *T. bryosalmonae-*exposed posterior kidney of brown trout

### Validation of transcriptional regulation

**Fig. 5:**
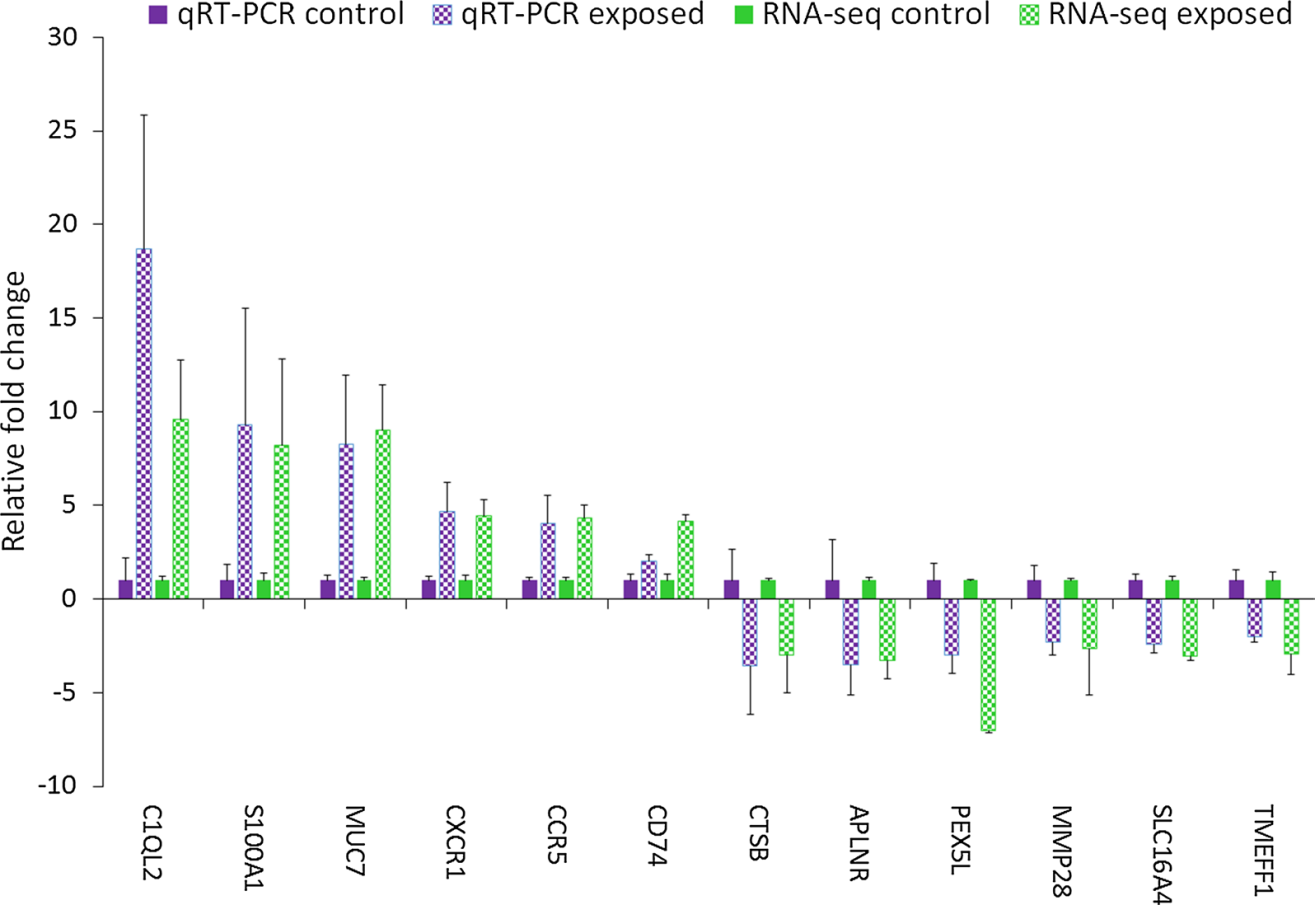
Validation of RNA-seq analysis by qRT-PCR on selected genes. The gene expression values are represented as relative fold change (mean ± SEM) of *Tetracapsuloides bryosalmonae-*exposed group compared to the unexposed control group (*n* = 6). The relative gene expression represented as the fold increase or decrease of the exposed group compared to the unexposed control group were calculated using 2^−ΔΔCt^ method and the mean expression levels were adjusted to one

**Fig. 6:**
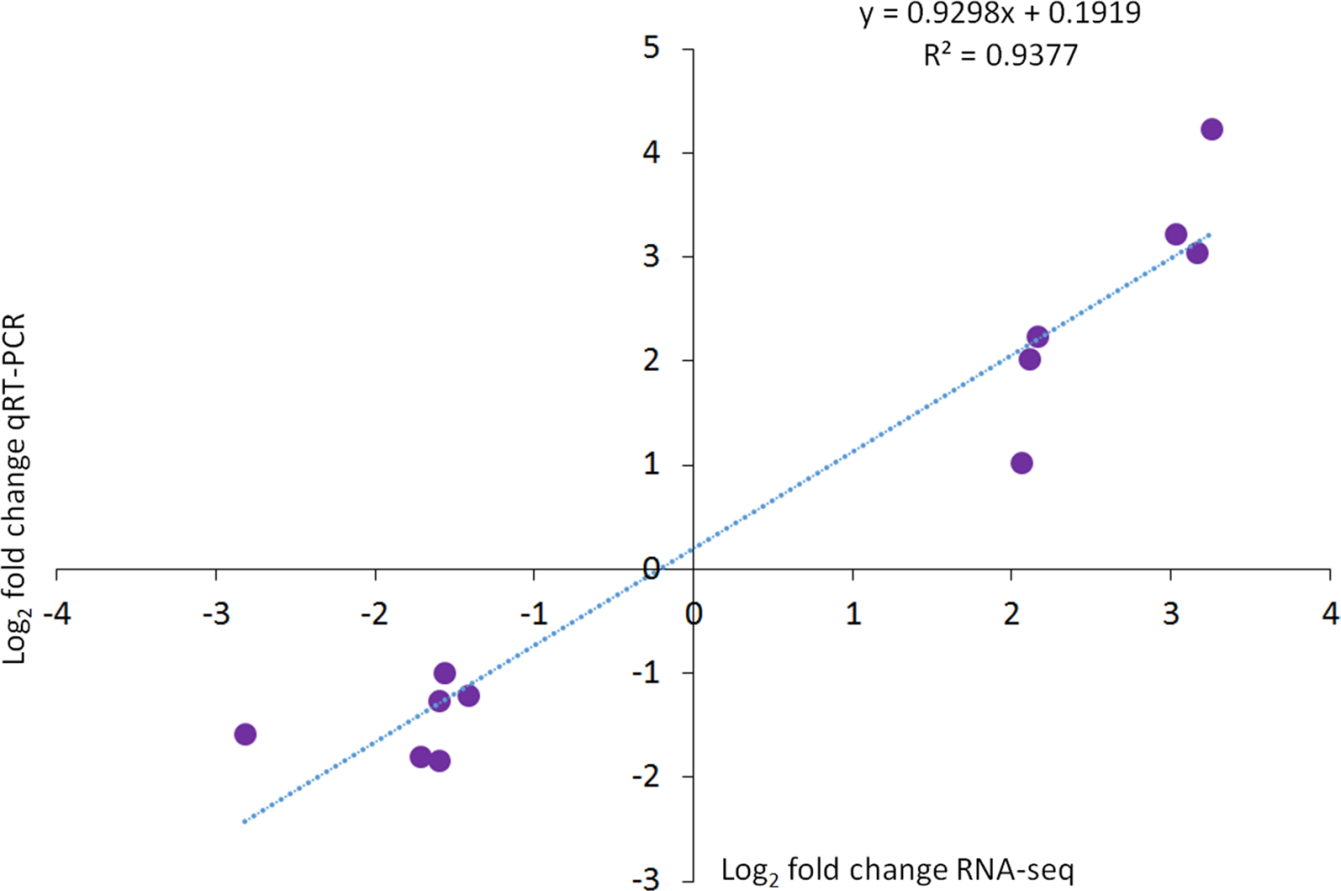
Linear regression plot between RNA-seq and qRT-PCR. The log_2_ fold change values for the RNA-seq and qRT-PCR are plotted along with the linear fit line showing a significant Pearsonʼs correlation coefficient *r*_(10)_ = 0.968, *P* < 0.0001 and a coefficient of determination *R*^2^ = 0.937

All selected DEGs were differentially up or downregulated in the exposed samples, relative to the unexposed control samples in qRT-PCR, that is, C1QL2 (*t*_(5.01)_ = − 4.2, *P* = 0.007), S100A1 (*t*_(5.04)_ = 2.58, *P* = 0.04), MUC7 (*t*_(5.0)_ = − 2.6, *P* = 0.04), CXCR1 (*t*_(5.07)_ = − 3.06, *P* = 0.02), CCR5 (*t*_(5.05)_ = − 2.67, *P* = 0.04), CD74 (*t*_(6.7)_ = − 2.72, *P* = 0.03), CTSB (*t*_(9.8)_ = 8.01, *P* < 0.0001), APLNR (*t*_(8.1)_ = 3.45, *P* = 0.008), PEX5L (*t*_(9.9)_ = 3.1, *P* = 0.01), MMP28 (*t*_(5.76)_ = 3.17, *P* = 0.02), SLC16A4 (*t*_(9.9)_ = 5.65, *P* = 0.02) and TMEFF1 (*t*_(6.4)_ = 3.26, *P* = 0.0002). As shown in Fig. 5, the relative gene expression levels of selected genes measured by qRT-PCR were consistent with the corresponding RNA-seq transcriptome data. A significant positive correlation (*r*_(10)_ = 0.968, *P* < 0.0001) between the log_2_ fold change values of RNA-seq and qRT-PCR affirms the reliability and reproducibility of the RNA-seq analysis (Fig. 6).

## Discussion

Fishes are lower vertebrates and their defence systems against the invading pathogens are comparable with those in mammalian higher vertebrates [38]. However, there are a few differences in the immune system between fish and mammals. Fish do not have bone marrow and lymph nodes; instead, the kidney functions as a major lymphoid organ in teleost fish and serves as both primary and secondary lymphoid organ [39]. *Tetracapsuloides bryosalmonae* is an interesting parasite that can develop and persist chronically in the kidney of brown trout. The PKD-survived brown trout acts as a reservoir of *T. bryosalmonae* and shed the infectious parasite *via* urine for its lifetime [18]. The host-parasite coevolution has come to a point where both brown trout and *T. bryosalmonae* can coexist together; however, the parasite has not coevolved with the dead-end rainbow trout host [17]. Previous studies based on PCR and qRT-PCR analyses have examined cellular responses and immune genes in the kidney of rainbow trout infected with *T. bryosalmonae* [4, 19–22, 25, 40] but only limited information is available about the brown trout [24, 26]. The present study attempted to explore the global transcriptome of posterior kidney of brown trout to gain insights into the host molecular machineries during PKD.

Recent studies on transcriptome analysis in the posterior kidney of rainbow trout by Bailey et al. [23] at a late phase of infection (130 days post-infection) identified only 280 DEGs in the PKD affected group. Among the 280 DEGs, 36 were identified as immune-related genes and interestingly, 35 of them were downregulated, except for single Ig IL-1-related receptor gene. At this late stage of parasite development, rainbow trout host exhibited a trade-off by reducing immune actions and increasing metabolic processes to recover from the disease [23]. In our study even after observing stringent selection criteria (adjusted *P* < 0.01), we identified 1169 DEGs, of which 864 were upregulated and 305 genes were downregulated in the posterior kidney of brown trout. Furthermore, GO analysis revealed that most of the upregulated genes were associated with the regulation of immune system process. This high number of DEGs in the kidney of brown trout may be due to the active immune response against the parasite.

### Host immune response

Our transcriptomic analysis identified an intense immune response in the posterior kidney of brown trout against *T. bryosalmonae*, characterized by the involvement of various elements of both innate and adaptive immune system. Previous investigations on PKD suggest proliferation of lymphocytes, reduction of the myeloid cell population, and complex interaction of Th1 and Th2 cells during pathogenesis in the affected rainbow trout [4, 20–22, 26]. In our study, cytokines and associated genes were clearly overrepresented among the upregulated DEGs including chemokines, interferons, interleukins, tumor necrosis factors, transforming growth factors and colony stimulating factors.

Chemokines are crucial elements of the innate immune system involved in chemo-attraction and trafficking of various immune cells to the site of infection and serve as a bridge between innate and adaptive defence system [41]. Neutrophil chemotactic factor CXCL8, had a peak expression at 50 days post-exposure in brown trout kidney in response to *T. bryosalmonae* [26]. In the present study, chemokines and their receptors (CXCL12, CXCL13-like, CXCR1-like, CXCR5-like, CCR5-like, CCL4, and CCL11-like) were predominantly upregulated in the posterior kidney of brown trout, except for CXCR3-like (− 2.4-fold) gene. In contrast, CCL4, CXCF1A and CCL13 were downregulated in the posterior kidney of rainbow trout during the late phase of PKD [23]. Previous studies suggest that chemokine molecules were activated in fish during parasitic infections such as *Ichthyophthirius multifiliis* [42] and *Cryptocaryon irritans* [43]. Similarly, CCL19 known for its chemotactic properties was upregulated in the head kidney of the turbot (*Scophthalmus maximus*) infected with the intestinal myxozoan parasite *Enteromyxum scophthalmi* [44]. B cells (IgM^+^ and IgT^+^) isolated from the intestine of rainbow trout infected with *Ceratomyxa shasta* showed expression of chemokine receptor CCR7 [45].

Interferons (IFNs) are cytokines responsible for mobilizing the host defence system against the invading pathogens. IFN-α/β receptor subunits (IFNAR1-like and IFNAR2-like) and interferon-γ receptor subunit (IFNGR1) were upregulated in the posterior kidney of brown trout during *T. bryosalmonae* proliferation. Type I IFNs (IFN-α and IFN-β) binds to IFNAR1/IFNAR2 receptors, whereas Type II IFN (IFN-γ) binds to IFNGR1/IFNGR2 receptors, but both signal their respective Janus-activated kinases (JAK) molecules initiating a cascade of immune action against invading pathogens [46]. SOCS proteins can negatively regulate IFN pathway and associated inflammatory response in fish. SOCS-1 has been demonstrated as a potential suppressor of IFN pathway and associated JAK-STAT signaling pathway in Atlantic salmon [47]. In our study, we observed upregulation of SOCS-1-like gene (3.6-fold) in the posterior kidney of *T. bryosalmonae*-exposed brown trout, which may have a negative effect on IFN production during PKD. Higher expression of SOCS-1 and SOCS-3 may play an important role in immunosuppression in rainbow trout, which facilitates host evasion process of *T. bryosalmonae* [20, 25, 48].

Interleukin (IL12B-like) and interleukin receptors (IL12RB2-like, IL13RA2-like, IL21R-like and IL6ST-like) were upregulated in the posterior kidney of brown trout during *T. bryosalmonae* proliferation. However, interleukin molecules (IL2RB2, IL21R, IL6RA, IL12B, IL4/13A, IL-1RA and IL-1RII) were downregulated during late phase of PKD infection in rainbow trout [23]. IL12 indirectly promotes antiparasitic activity of macrophages and cytolytic function of natural killer cells [49]. Furthermore, IL-13 produced by Th2 cells is known for its defensive response against metazoan parasites [50]. This indicates that interleukins may play an active role in the brown trout against *T. bryosalmonae* development. However, in the present study downregulation of proinflammatory cytokine IL16-like (− 2.7-fold) was noticed in the posterior kidney. IL16 is known for its function in the modulation of T-cell response and chemoattraction of immune cells [49]. Furthermore, transforming growth factor beta induced protein (4.7-fold) was upregulated and tumour necrosis factor alpha induced protein 8 (− 2.2-fold) was downregulated in the kidney of brown trout in response to *T. bryosalmonae*.

IL12, IL16, IL21 and TNFα are pro-inflammatory markers whereas IL13 and TGFβ are markers of anti-inflammatory function. Interestingly, IL6 has both pro-inflammatory and anti-inflammatory functions [51]. Gorgoglione et al. [20] observed higher expression of anti-inflammatory genes (IL6, IL10, IL11, nIL-IF, SOCS1 and SOCS3) in *T. bryosalmonae-*infected rainbow trout. Additionally, these authors did not find any correlation between the expression of pro-inflammatory molecules (TNFα, IL1β and COX2 isoforms) and *T. bryosalmonae* prevalence. Similarly, Bailey et al. [26] also found un-responsiveness of pro-inflammatory cytokines (TNFα, IL1β and IFNγ) in PKD infected brown trout [26]. Taken together, PKD pathogenesis tends the inflammatory mechanism towards anti-inflammatory phenotype in trout [52].

T cells have a crucial role in the immune defence against the invading pathogens and act as an effector by directly killing the infected cell or coordinating other immune cells against the invading pathogen [53]. T cells are characterized based on the surface T-cell receptors such as αβ-T cells and γδ-T cells. We identified a strong positive regulation and proliferation of αβ-T cells in the posterior kidney of brown trout during *T. bryosalmonae* development. αβ-T cells can recognize parasite-derived antigens presented by major histocompatibility complex (MHC) and a noticeable upregulation of MHC class I alpha chain (3075.7-fold) and MHC class II DP beta 2 (2.4-fold) were observed in the posterior kidney of brown trout in our study. Similar upregulation of MHC class I molecule was observed in the spleen of rainbow trout during *Yersinia ruckeri* infection [54]. However, experimental infection with the intestinal myxozoan parasite *E. scophthalmi* in turbot resulted in downregulation of the MHC class I molecule, which was attributed to the host immune evasion by the parasite [55]. Signatures of both Th1 (T-bet, IFNγ, TNFα and IL-2) and Th2 (GATA3, IL4/13A, IL10 and FOXP3) cells were differentially regulated during *T. bryosalmonae* infection in rainbow trout and brown trout [20, 26, 40]. Interestingly, we also found upregulation of marker of regulatory T cell [CD3E] (5.6-fold) in brown trout exposed to *T. bryosalmonae*. This suggests that PKD pathogenesis drive towards a dysregulated Th cell activity in salmonids [20, 26] possibly associated with the chronic infection nature of this disease [56].

Complement system is a major effector system of innate and acquired immunity which aids in the pathogen clearance mechanism [57]. In mammals, the complement system has been studied extensively; however, this is least studied in teleost fish. Complement associated molecules like C4A (15.4-fold), C1QL2 (9.5-fold) and CR2-like (3.2-fold) were upregulated whereas C8G (− 5.7-fold) was downregulated in the posterior kidney of brown trout in response to *T. bryosalmonae*. The role of complement system against parasitic infection in fish has been documented. In salmonids, activation of the alternative pathway of the complement system was shown to act against the monogenean parasites, *Gyrodactylus salaries* [58] and *Discocotyle sagittata* [59]. Similarly, parasitic ciliate *I. multifiliis* infection led to the higher expression of C3 in the skin mucus and lymphoid organs of rainbow trout [60], and the proteins involved in pathogen recognition and complement activation [61]. C8 is a part of membrane attack complex and the downregulation of C8G in the present study may be attributed to the parasite-induced modulation to escape host immune response. *Tetracapsuloides bryosalmonae* proliferation in the kidney of brown trout has generated a complex interaction of immune responses and further detailed studies are essential to understand their specific roles during host-parasite interaction.

### Calcium-binding proteins

In humans and other higher vertebrates, regulation of intracellular calcium is associated with homeostasis and is regulated by calcium-binding proteins. These proteins have clinical importance in inflammation, cancer, neurology, allergy, cardiomyopathy and immune response [62]. Nevertheless, calcium-binding proteins and their importance on homeostasis are least studied in fish. In the present study, two calcium-binding proteins S100A1 (8.1-fold) and calretinin CALB2 (14.8-fold) were upregulated in the posterior kidney of brown trout in response to *T. bryosalmonae*. Calretinin is a vitamin D-dependent calcium-binding protein known to interact with cytoskeletal components [63], whereas S100 is involved in the modulation of specific signal transduction pathway, control of cell growth and its proliferation [64]. In contrast to our results, omics studies have revealed downregulation of S100 in the kidney of the gilthead sea bream (*Sparus aurata*) infected with the myxozoan parasite *Enteromyxum leei* [65]. Protein interaction experiments have identified active interaction between calcium-binding protein S100A9 in the kidney of brown trout and *T. bryosalmonae* [66]. This explains the importance of these calcium-binding proteins in the kidney of brown trout during PKD pathogenesis and further experiments are required to explore their specific roles.

### Apoptosis

Parasites have been identified to modulate the host apoptotic regulatory system to facilitate their invasion, differentiation, and replication in their host. Intracellular parasites can inhibit apoptosis in the cells where they dwell, whereas, extracellular parasites are known to induce apoptosis in the host immune cells that hinder their evasion or the cells that have components of their diet [67]. Host genes involved in the regulation of apoptosis were differentially expressed in our study. Pronounced upregulation of CASP14-like (1704.4-fold), BCL2L11 (4.0-fold), BCL7B (2.2-fold), and BIRC2 (2.3-fold) were observed in brown trout during *T. bryosalmonae* proliferation. However, BIRC2 is known for its role in the inhibition of apoptosis. Similarly, in previous studies CASP14 precursor protein was identified in the kidney of PKD-affected brown trout kidney by antibody-based protein purification followed by ESI-MS [66]. It is important to highlight that the inducers of apoptosis, cathepsin molecules CTSB (− 3.0-fold), CTSD (− 3.6-fold), and CTSK (− 6.5-fold) were downregulated in the posterior kidney of parasite-exposed brown trout. Cathepsins are multifunctional proteolytic enzymes involved in various immune processes including antimicrobial activity [68]. Our results suggest that *T. bryosalmonae* proliferation strongly influence apoptotic process in brown trout. Similar differential expression of host apoptosis molecules was also observed in the kidney transcriptome of rainbow trout during PKD recovery phase [23].

### Endopeptidase regulatory activity

Molecules involved in endopeptidase regulatory activity had a clear downregulation pattern in the posterior kidney of parasite-exposed brown trout. Genes involved in the negative regulation of endopeptidase regulatory activity (A2M-like, AMBP-like, GAPDH and SERPINH1) were downregulated in brown trout exposed with *T. bryosalmonae*. A2M is a protease inhibitor known to inhibit the proteases produced by parasites during host infection process. A2M is reported to have defense role against the fish parasites such *Cryptobia salmositica* in salmonids [69], and *Trypanoplasma borreli* and *I. multifiliis* in common carp [70]. In higher vertebrates, apart from anti-protease activity A2M is known for its role in the activation and proliferation of macrophages [71], involvement in the inhibition of clotting cascade [72], and mediate T-cell proliferation [73]. Moreover, A2M can bind with hormones, cytokines, endopeptidases, histones, mitogens, and various ions [74]. In our transcriptome results, A2M-like gene was downregulated (− 7.8-fold) in the posterior kidney of brown trout exposed to *T. bryosalmonae*. This suggests that *T. bryosalmonae* may have evolved to modulate and reduce the activities of A2M protease inhibitors in the kidney of brown trout to facilitate parasite proliferation and release into the aquatic environment *via* urine.

### Collagen catabolic process

Genes associated with collagen catabolic process (CTSB, CTSD, CTSK-like, MMP13, MMP28-like and VSIR) were downregulated in the the posterior kidney of brown trout in response to *T. bryosalmonae*. MMP13 and MMP28 represent matrix metalloproteinases family of genes and have a major role in host extracellular matrix degradation and remodeling. MMP13 has been suggested to play a crucial role in inflammatory response of Atlantic salmon during salmon louse (*Lepeophtheirus salmonis*) parasitic infection. Activation of MMP9 and MMP13, and the downregulation of extracellular structural protein, prolonged the wound healing process at the site of salmon louse attachment [75]. Bailey et al. [23] reported upregulation of collagen catabolic genes such MMP16 and CTSB in the kidney of rainbow trout during late phase of PKD infection. The upregulation of host collagen catabolic protease genes might influence the sporogenesis of *T. bryosalmonae* in rainbow trout dead-end host. However, we could not find any statistically significant (≥ |2.0| fold and adjusted *P*-value < 0.01) upregulation of collagen catabolic genes in the kidney of brown trout during active phase of *T. bryosalmonae* proliferation. Taken together, the downregulation of collagen catabolic proteases in brown trout might be a possible trade-off between host and parasite, by reducing the host collagen catabolic proteases activity against the parasite and accelerating the tissue repair process in the kidney. This may facilitate brown trout and *T. bryosalmonae* to coexist together. We reported previously that the kidney of brown trout was found recovered after five years of *T. bryosalmonae* exposure that could excrete viable parasite spores infectious to bryozoans [18]. Further investigations are required to check whether any protease inhibitors of *T. bryosalmonae* are upregulated during developmental stages in the kidney of brown trout.

### Connective tissue development

Among the genes related to connective tissue development, special attention must be paid to connective tissue growth factor CCN2 of CCN gene family, which enhances cell proliferation, myofibroblast differentiation, and extracellular matrix production. In higher vertebrates dysregulation of CCN2 may result in the inhibition of tissue repair process, which leads to excessive scarring and fibrosis [76]. *Trypanosoma cruzi* infection in humans interfered with host fibrogenic response and resulted in the downregulation of CCN2 in foreskin fibroblast cells [77]. Similarly, in the present study, we identified a downregulation of CCN2-like (− 3.2-fold) gene in the kidney and correlated with the tissue damage in the kidney during parasite development.

### Host-parasite coexistence

From the present study and previous works, it is evident that both rainbow trout and brown trout exhibits a strong immune action against *T. bryosalmonae* during the active infection phase [20–22]. However, downregulation of immune response was noticed at the late recovery phase of *T. bryosalmonae* infection in rainbow trout [23]. In spite of a strong defence response from both of its hosts, *T. bryosalmonae* could accomplish sporogenesis in brown trout, but not in rainbow trout [17]. Furthermore, Kumar et al. [24] described differences in the pattern of expression between brown trout and rainbow trout during active *T. bryosalmonae* infection, particularly in host genes involved in cell proliferation, cell growth, endocytic pathway, anti-inflammatory and humoral immune responses. *Tetracapsuloides bryosalmonae* has evolved to employ certain unknown mechanism to survive the immune response of brown trout. In addition, the downregulation of host proteases observed in the present study may be due to protease inhibitors produced by *T. bryosalmonae.* It would be interesting to study the aspects of parasite counter-mechanism against the brown trout immune response and the role of parasite protease inhibitors, in *T. bryosalmonae* sporogenesis in brown trout. Further study is needed to explore *T. bryosalmonae* transcriptome during active development in brown trout, which can provide detailed insights about the molecular strategies adopted by the parasite to facilitate its coexistence with brown trout host.

## Conclusions

To our knowledge, this is the first transcriptome profiling of the posterior kidney of brown trout during the active phase of *T. bryosalmonae* proliferation. Functional annotation of differentially expressed genes showed that the upregulated genes were associated with molecular functions such as cytokine receptor activity, binding identical proteins, peptides, enzymes, and cytoskeleton proteins. Whereas, the downregulated genes were associated with endopeptidase regulator activity, proteoglycan and spectrin binding, and symporter activity. The downregulation of host collagen catabolic proteases might be a possible trade-off between brown trout and *T. bryosalmonae*. Our study provides new insights into the brown trout-*T. bryosalmonae* interaction, particularly on the host immune components that acted against the parasite, and on the host genes modulated by the parasite to establish themselves in the host. However, further studies are needed to characterize the evasion strategy adopted by the parasite to reach the target organ kidney in the salmonid host.

## Supplementary information

**Additional file 1: Table S1.** List of quantitative qRT-PCR primers used in this study.

**Additional file 2: Table S2.** List of all significant differentially expressed up- and downregulated genes.

**Additional file 3: Table S3.** Gene ontology terms enriched by up- and downregulated genes separately.

**Additional file 4: Figure S1.** Overview of biological processes of upregulated genes. **Figure S2.** Specific functional GO terms of biological processes of upregulated genes. **Figure S3.** Overview of cellular components of upregulated genes. **Figure S4.** Specific functional GO terms of cellular components of upregulated genes. **Figure S5.** Overview of molecular functions of upregulated genes. **Figure S6.** Specific functional GO terms of molecular functions of upregulated genes.

**Additional file 5: Figure S7.** Overview of biological processes of downregulated genes. **Figure S8.** Specific functional GO terms of biological processes of downregulated genes. **Figure S9.** Overview of cellular components of downregulated genes. **Figure S10.** Specific functional GO terms of cellular components of downregulated genes. **Figure S11.** Overview of molecular functions of downregulated genes. **Figure S12.** Specific functional GO terms of molecular functions of downregulated genes.

**Additional file 6: Table S4.** List of all pathways mapped in KEGG pathway analysis.

## Data Availability

All raw sequence data have been submitted to the NCBI Short Read Archive (SRA) portal under NCBI Bioproject ID PRJNA542491.

## Abbreviations

APLNR: apelin receptor A-like
C1QL2: complement C1q like-2
CCR5: C-C chemokine receptor type 5-like
CD74: H-2 class II histocompatibility antigen gamma chain-like
CTSB: cathepsin-B
CXCR1: C-X-C chemokine receptor type 1-like
DEGs: differentially expressed genes
GO: gene ontology
H&E: hematoxylin and eosin
IFNs: interferons
IHC: immunohistochemistry
JAK: Janus-activated kinases
KEGG: Kyoto Encyclopedia of Genes and Genomes
MHC: major histocompatibility complex
MMP28: matrix metallopeptidase 28
MUC7: mucin-7
PEX5L: PEX5-related protein-like
PKD: proliferative kidney disease
qRT-PCR: quantitative real time PCR
RNA-seq: RNA sequencing
SLC16A4: solute carrier family 16 member 4
SOCS: suppressors of cytokine signaling
SPF: specific pathogen free
TMEFF1: tomoregulin-1-like
TNFα: tumour necrosis factor alpha
wpe: weeks post-exposure
