# Supplementary material for: Transcriptome profiling of posterior kidney of brown trout, Salmo trutta, during proliferative kidney disease
Source: Parasit Vectors. 2019 Nov 29;12:569. doi: 10.1186/s13071-019-3823-y (PMC6884850; doi:10.1186/s13071-019-3823-y)
Supplement: Supplementary file 1 — Additional file 1: Table S1. List of quantitative qRT-PCR primers used in this study. [file 13071_2019_3823_MOESM1_ESM.doc]

**Additional file 1: Table S1:** List of quantitative qRT-PCR primers used in this study

| **Primer code** | **Sequence (5`-3`)** | **Amplicon size**  **(bp)** | **Accession Number** |
| --- | --- | --- | --- |
| C1QL2 Forward | AGAAAGGAACAGCGGCATAC | 168 | GFIS01029709.1 |
| C1QL2 Reverse | ATTGTGGTTCCACCCAGTTG |
| S100A1 Forward | GTGGCCTGTAACGAGTTCTT | 163 | GFIS01064376.1 |
| S100A1 Reverse | TTGAAACATTTGCCTGTTTGTCA |
| MUC7 Forward | ACAGTCACCTGTAGAGTTGATAATA | 184 | GFIS01055908.1 |
| MUC7 Reverse | CAGAGGTCCACTTCTTTGGAG |
| CXCR1 Forward | GACACTCACCTTTCCTGAAGC | 188 | GFIS01047200.1 |
| CXCR1 Reverse | CAGCAAGCAGAAGAGTGTCT |
| CCR5 Forward | TTCCGCCTACTACGATGGTT | 175 | GFIS01036319.1 |
| CCR5 Reverse | CCTCCTACACTTCACCAGGA |
| CD74 Forward | ACGAAAAGACTCCCATGACG | 144 | GFIS01025489.1 |
| CD74 Reverse | TCCATCTGTCTCTTCAGGCT |
| CTSB Forward | TATAAGAACGGCCCAGTGGA | 180 | GFIS01025308.1 |
| CTSB Reverse | GTCTGTGTTCCAGGAGTTGG |
| APLNR Forward | GTTAAAGAGGGGAAAGTGTGTTTG | 187 | GFIS01001607.1 |
| APLNR Reverse | TCCCCGACAGCATTTGTCT |
| PEX5L Forward | GTGAGTGGAGATAGGCCTTG | 162 | GFIS01053470.1 |
| PEX5L Reverse | TGCTCGCCAAAATAGACCAC |
| MMP28 Forward | AGGCCATTATGTCTGTGCAA | 174 | GFIS01037459.1 |
| MMP28 Reverse | CCCGGCCCAGGTTTAATATG |
| SLC16A4 Forward | GATTCCAGGCTTAATGTCCCAG | 148 | GFIS01066512.1 |
| SLC16A4 Reverse | GCCGTCTAGAGGGTAGTTTC |
| TMEFF1 Forward | AATGGGGCCTTAGTTTTCTCTT | 184 | GFIS01041313.1 |
| TMEFF1 Reverse | CGCTGATAAATAGCATGAGTCCA |
| EF-1α Forward | AGACAGCAAAAACGACCCCC | 167 | HF563594 |
| EF-1α Reverse | AACGACGGTCGATCTTCTCC |
