# Supplementary material for: Transcriptome profiling of posterior kidney of brown trout, Salmo trutta, during proliferative kidney disease
Source: Parasit Vectors. 2019 Nov 29;12:569. doi: 10.1186/s13071-019-3823-y (PMC6884850; doi:10.1186/s13071-019-3823-y)
Supplement: Supplementary file 4 — Additional file 4: Figure S1. Overview of biological processes of upregulated genes. Figure S2. Specific functional GO terms of biological processes of upregulated genes. Figure S3. Overview of cellular components of upregulated genes. Figure S4. Specific functional GO terms of cellular components of upregulated genes. Figure S5. Overview of molecular functions of upregulated genes. Figure S6. Specific functional GO terms of molecular functions of upregulated genes. [file 13071_2019_3823_MOESM4_ESM.pptx]

## Slide 1
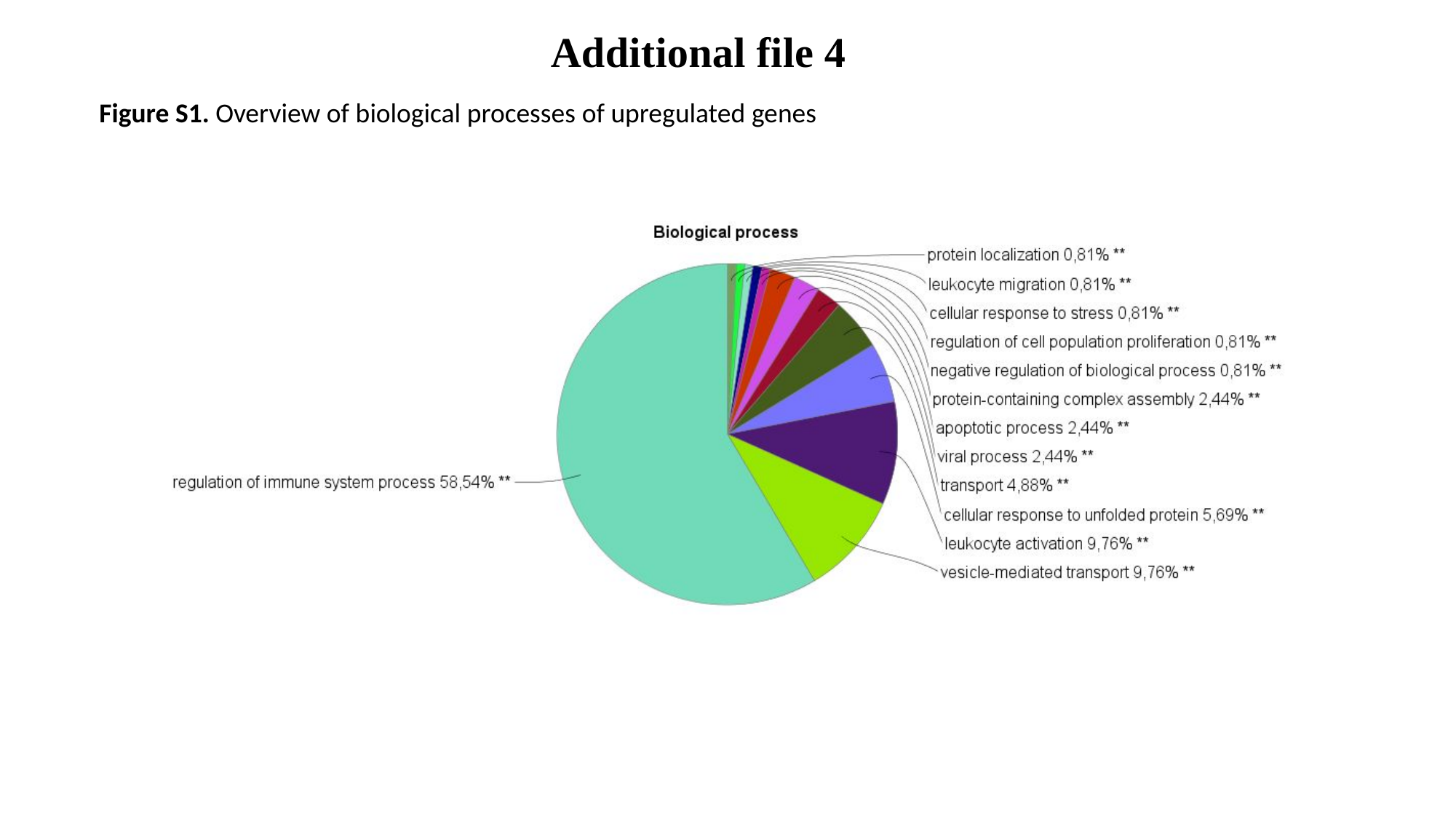

Additional file 4
Figure S1. Overview of biological processes of upregulated genes

## Slide 2
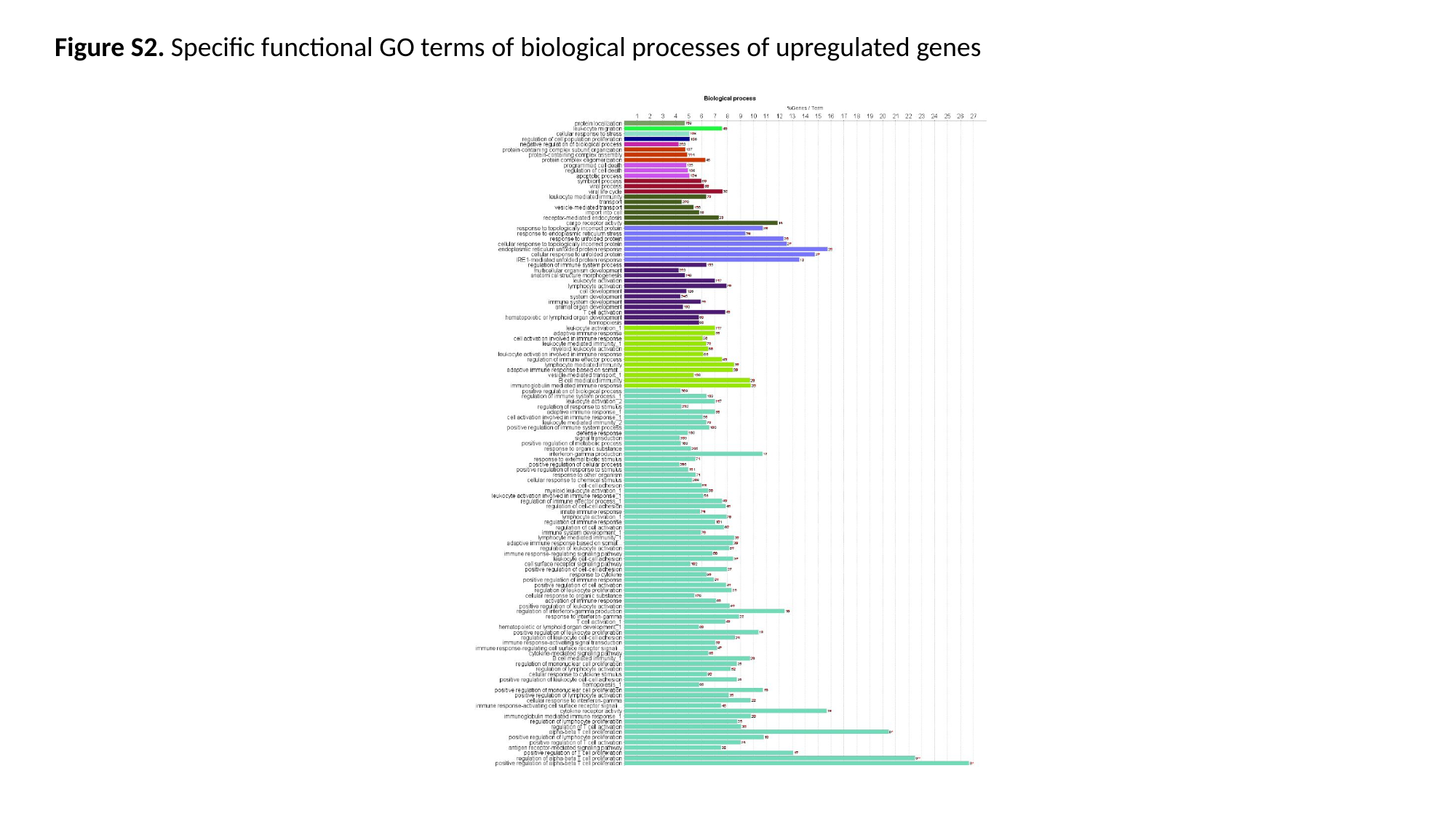

Figure S2. Specific functional GO terms of biological processes of upregulated genes

## Slide 3
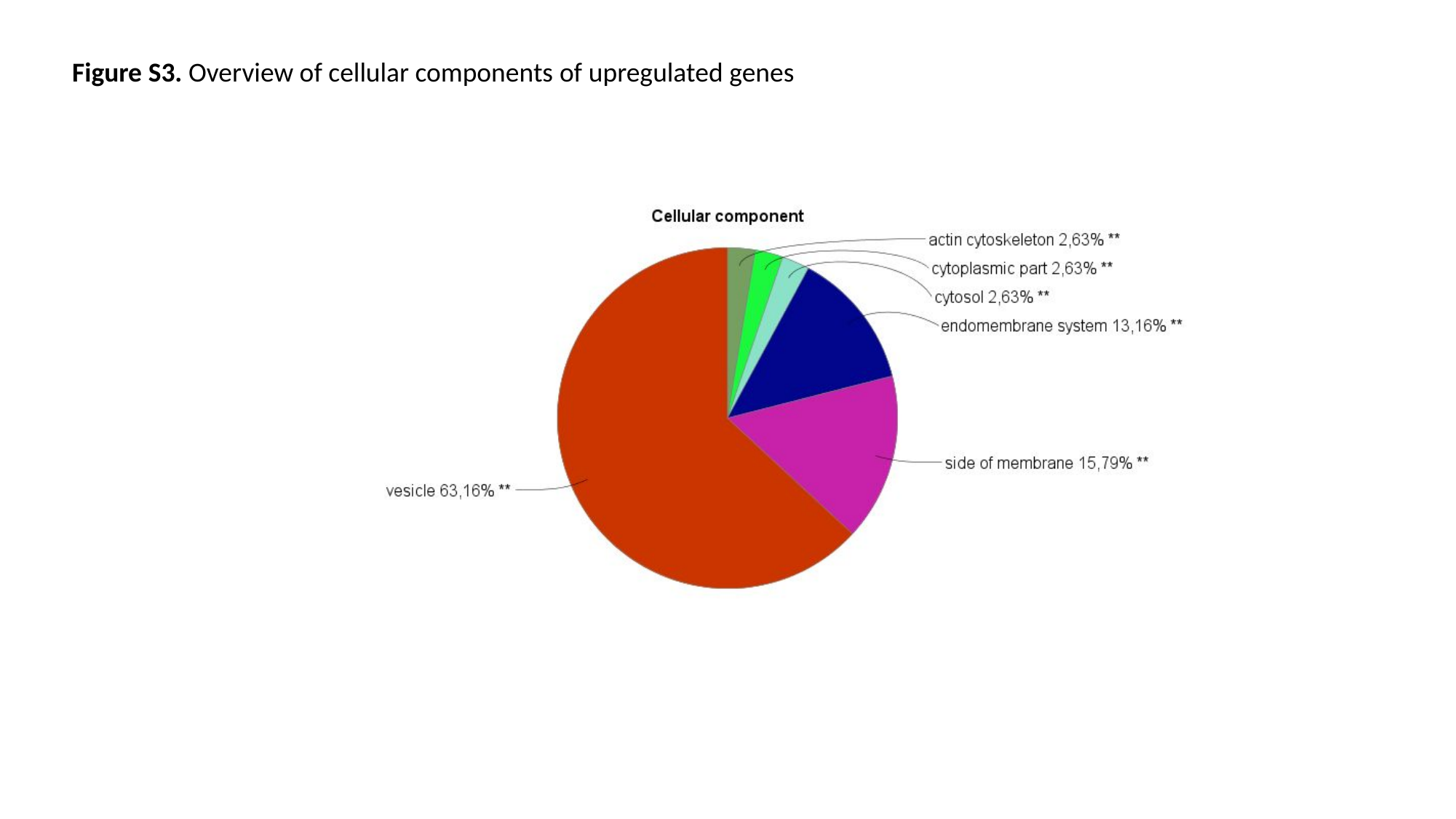

Figure S3. Overview of cellular components of upregulated genes

## Slide 4
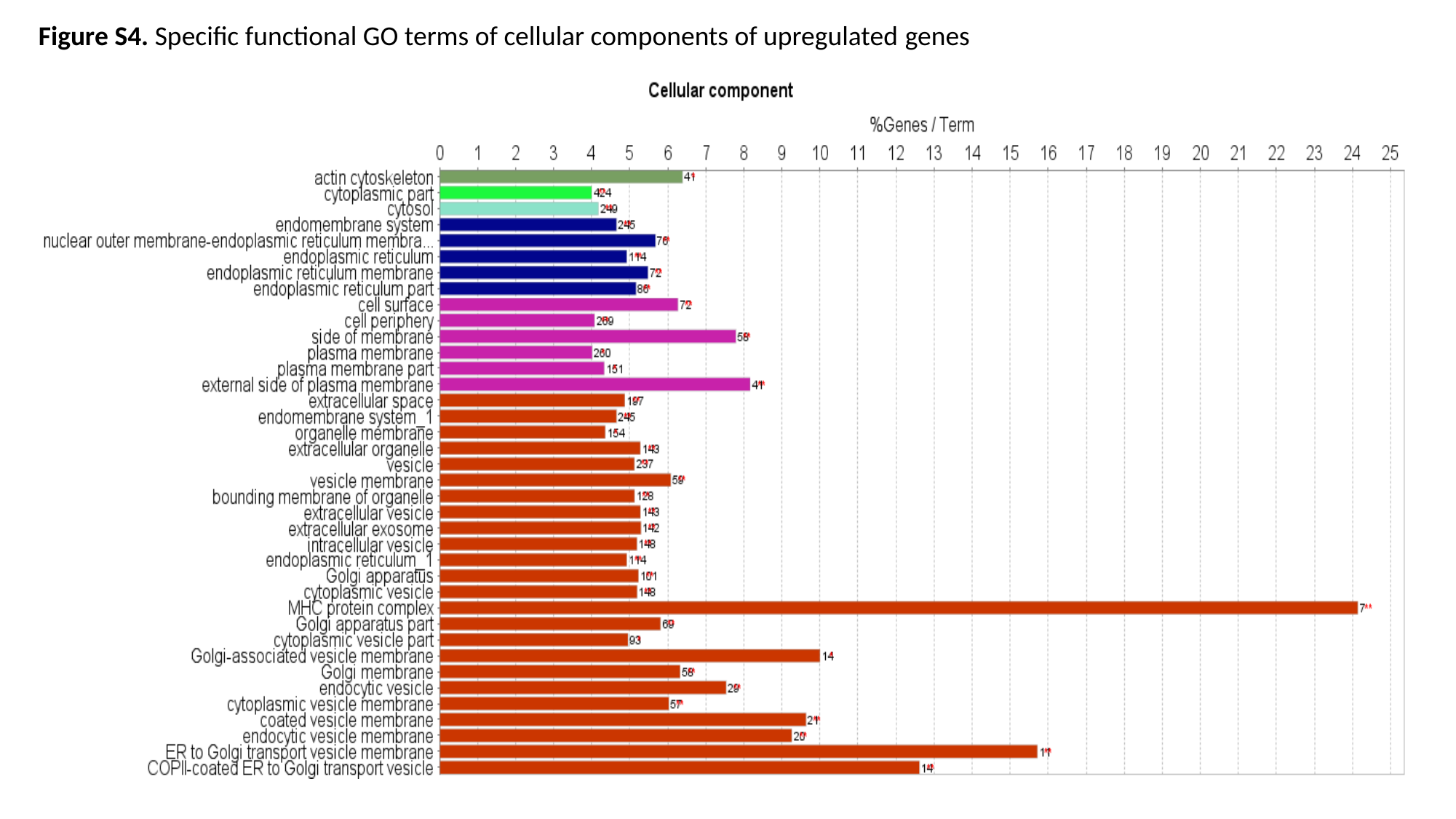

Figure S4. Specific functional GO terms of cellular components of upregulated genes

## Slide 5
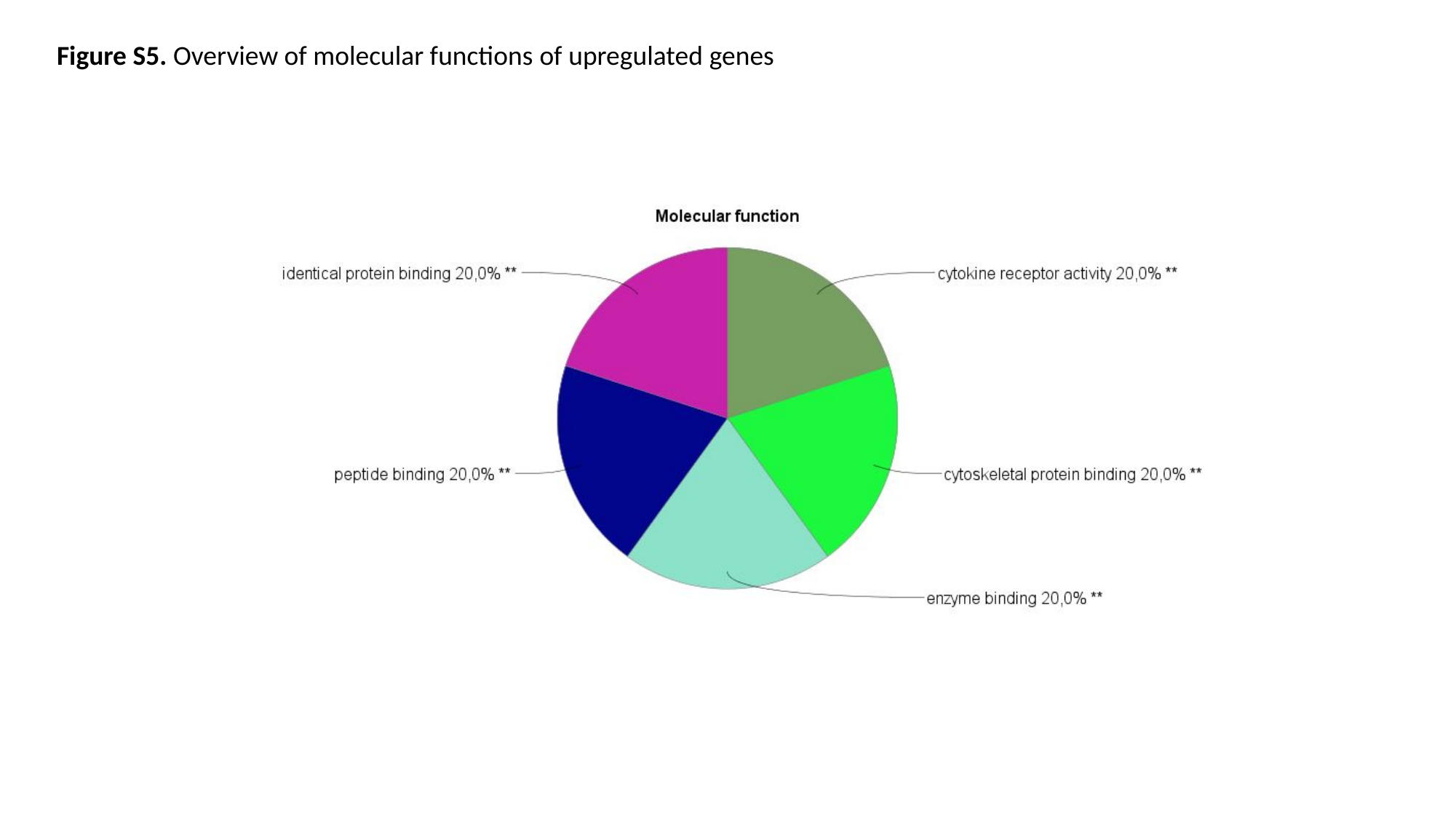

Figure S5. Overview of molecular functions of upregulated genes

## Slide 6
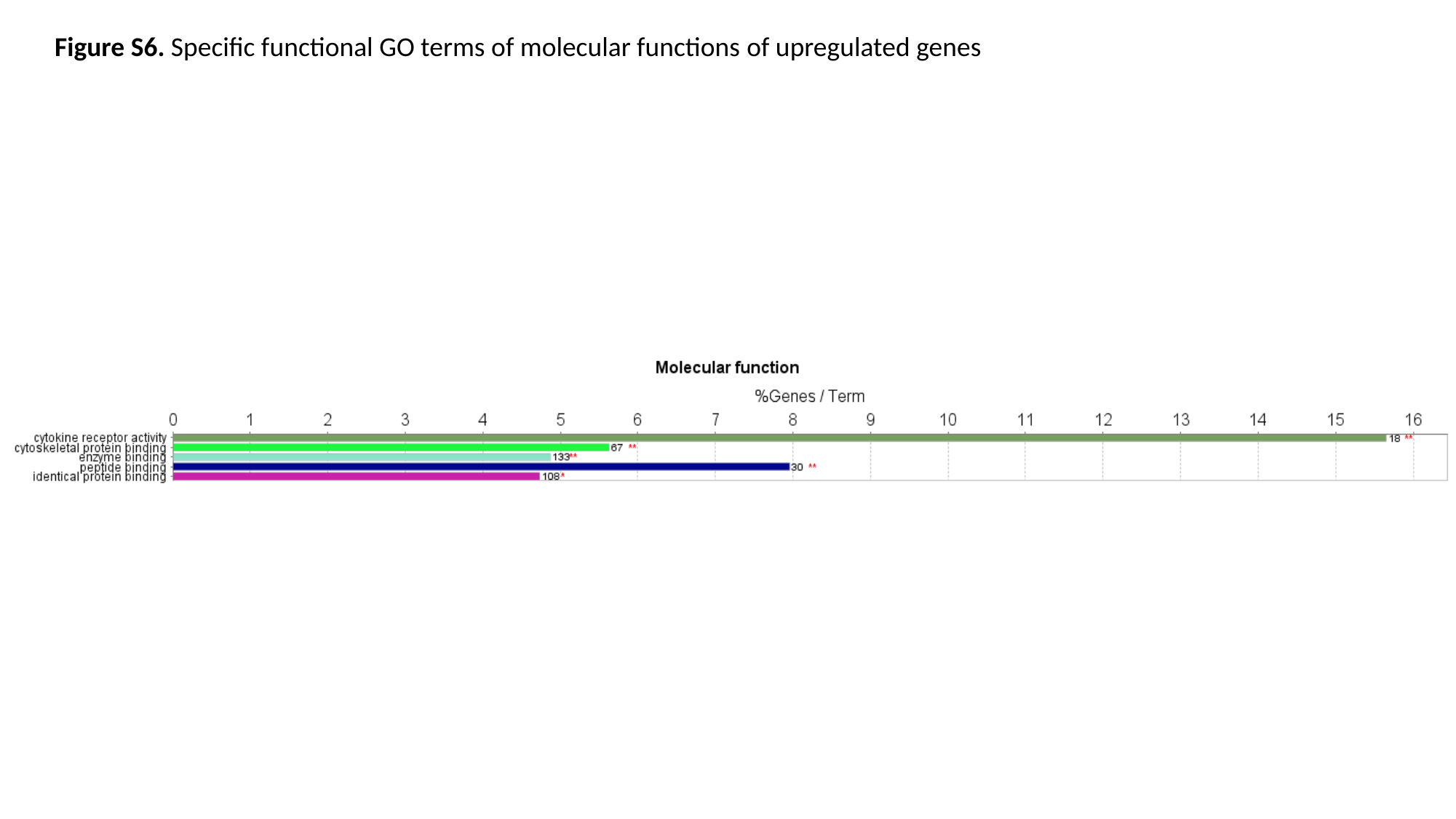

Figure S6. Specific functional GO terms of molecular functions of upregulated genes
