# Supplementary material for: Transcriptome profiling of posterior kidney of brown trout, Salmo trutta, during proliferative kidney disease
Source: Parasit Vectors. 2019 Nov 29;12:569. doi: 10.1186/s13071-019-3823-y (PMC6884850; doi:10.1186/s13071-019-3823-y)
Supplement: Supplementary file 5 — Additional file 5: Figure S7. Overview of biological processes of downregulated genes. Figure S8. Specific functional GO terms of biological processes of downregulated genes. Figure S9. Overview of cellular components of downregulated genes. Figure S10. Specific functional GO terms of cellular components of downregulated genes. Figure S11. Overview of molecular functions of downregulated genes. Figure S12. Specific functional GO terms of molecular functions of downregulated genes. [file 13071_2019_3823_MOESM5_ESM.pptx]

## Slide 1
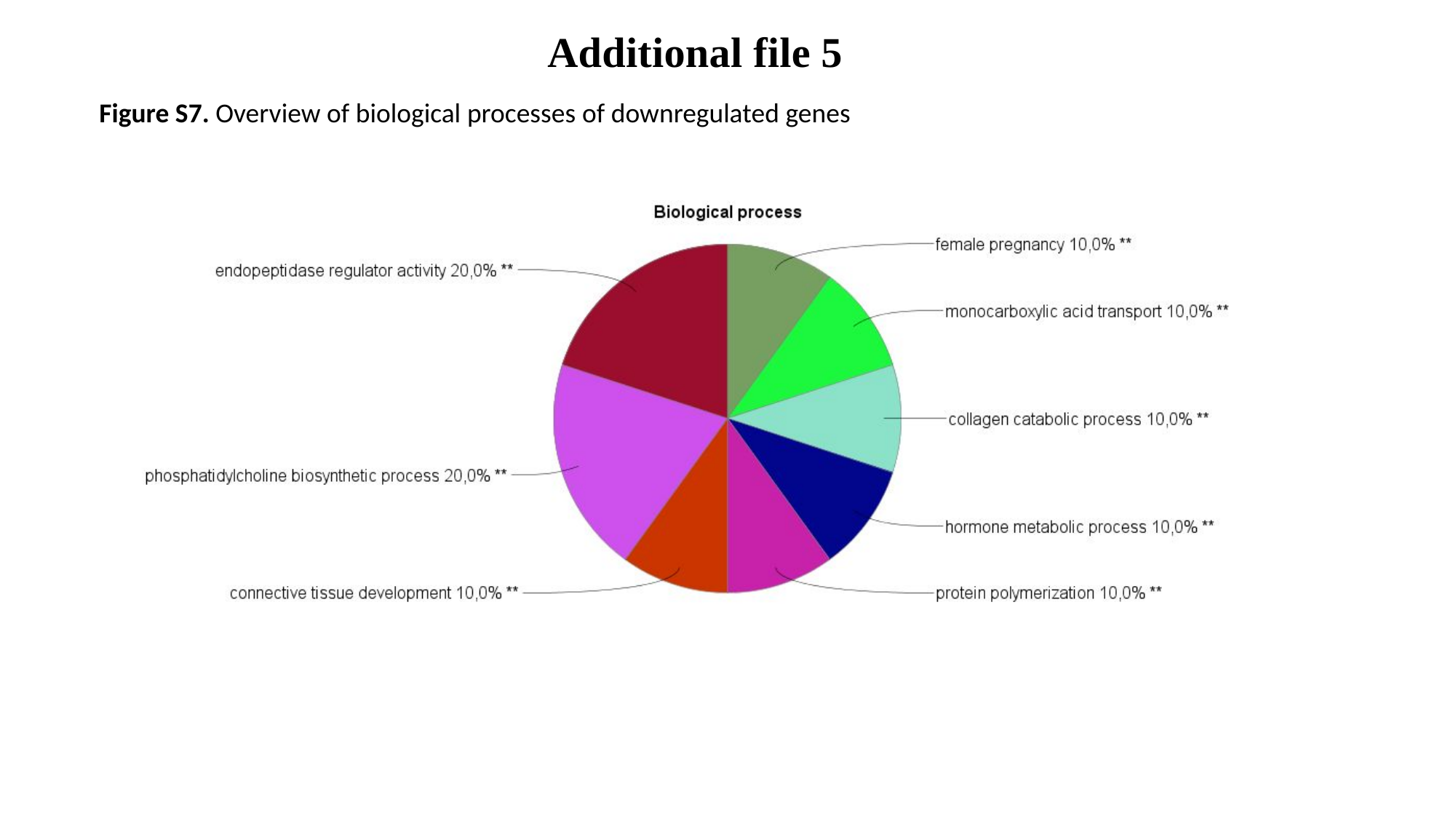

Additional file 5
Figure S7. Overview of biological processes of downregulated genes

## Slide 2
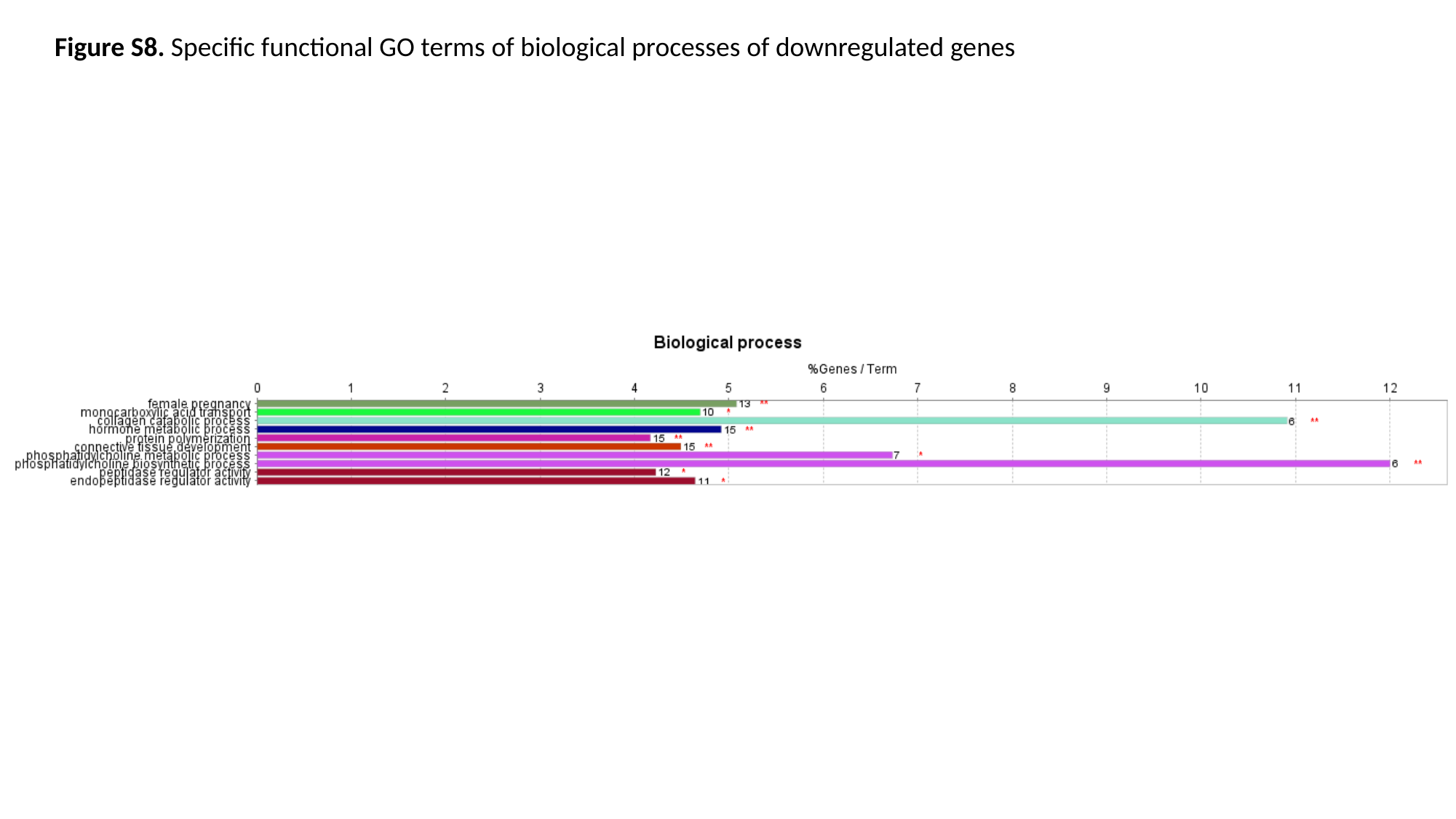

Figure S8. Specific functional GO terms of biological processes of downregulated genes

## Slide 3
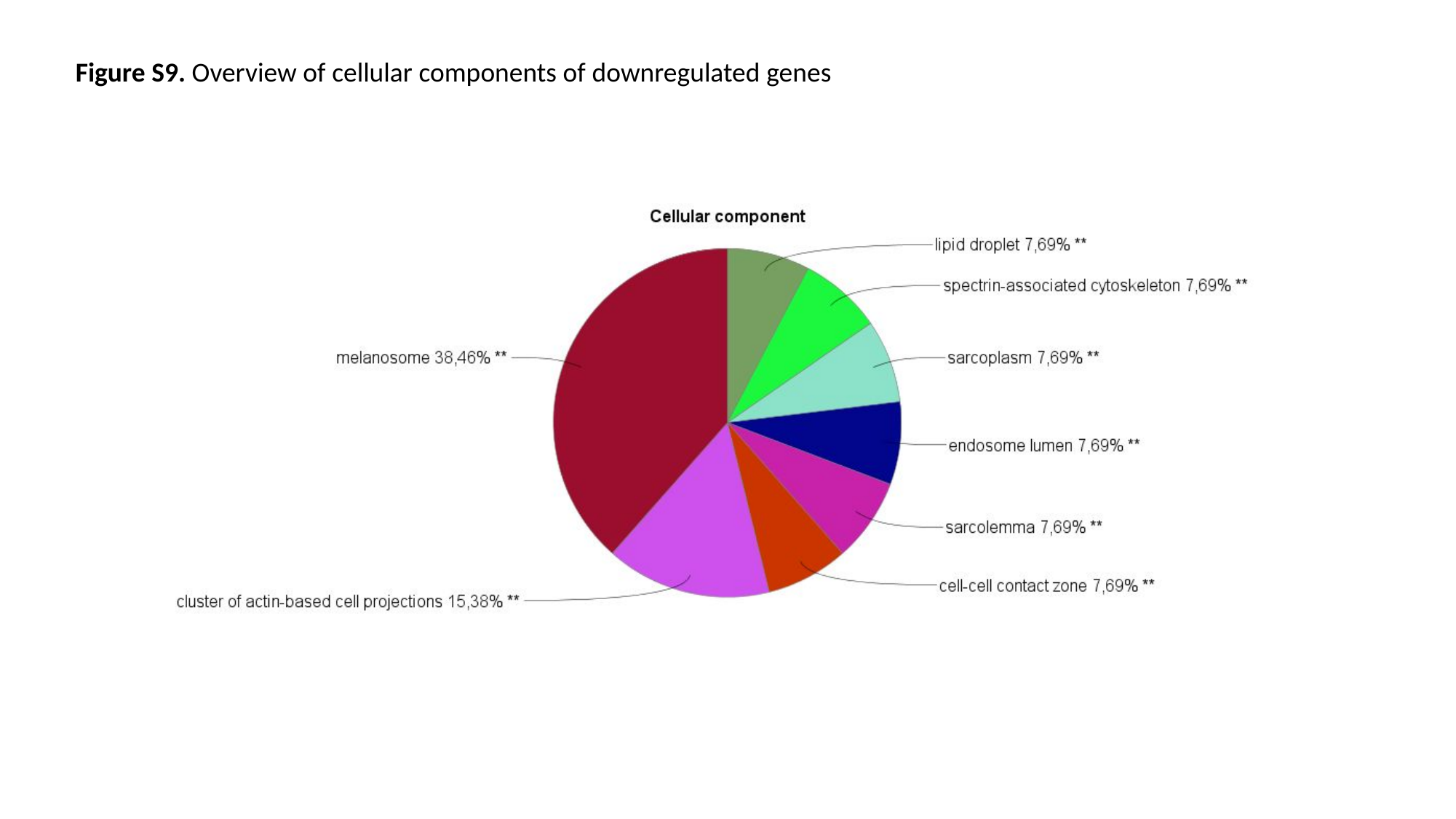

Figure S9. Overview of cellular components of downregulated genes

## Slide 4
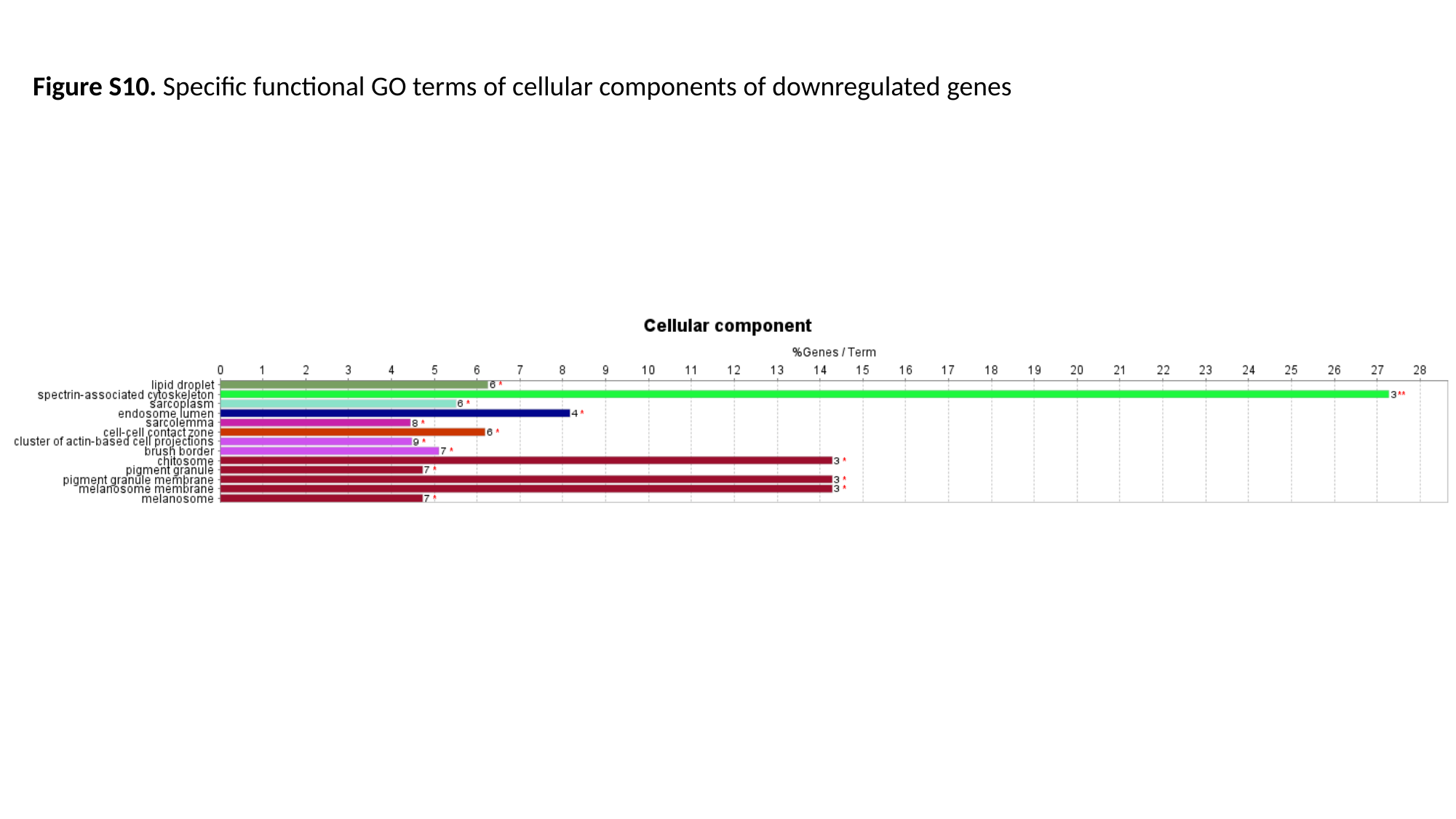

Figure S10. Specific functional GO terms of cellular components of downregulated genes

## Slide 5
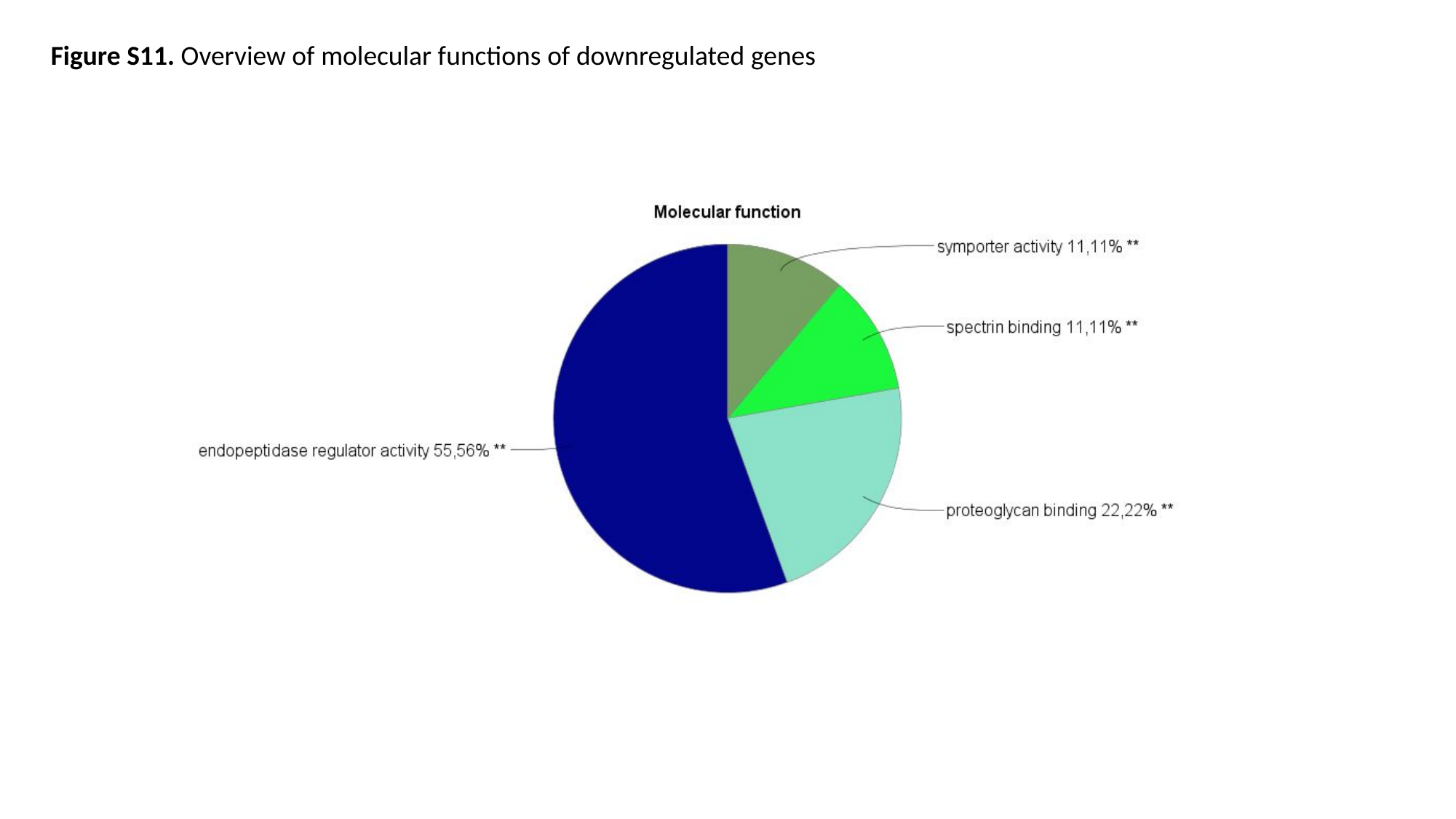

Figure S11. Overview of molecular functions of downregulated genes

## Slide 6
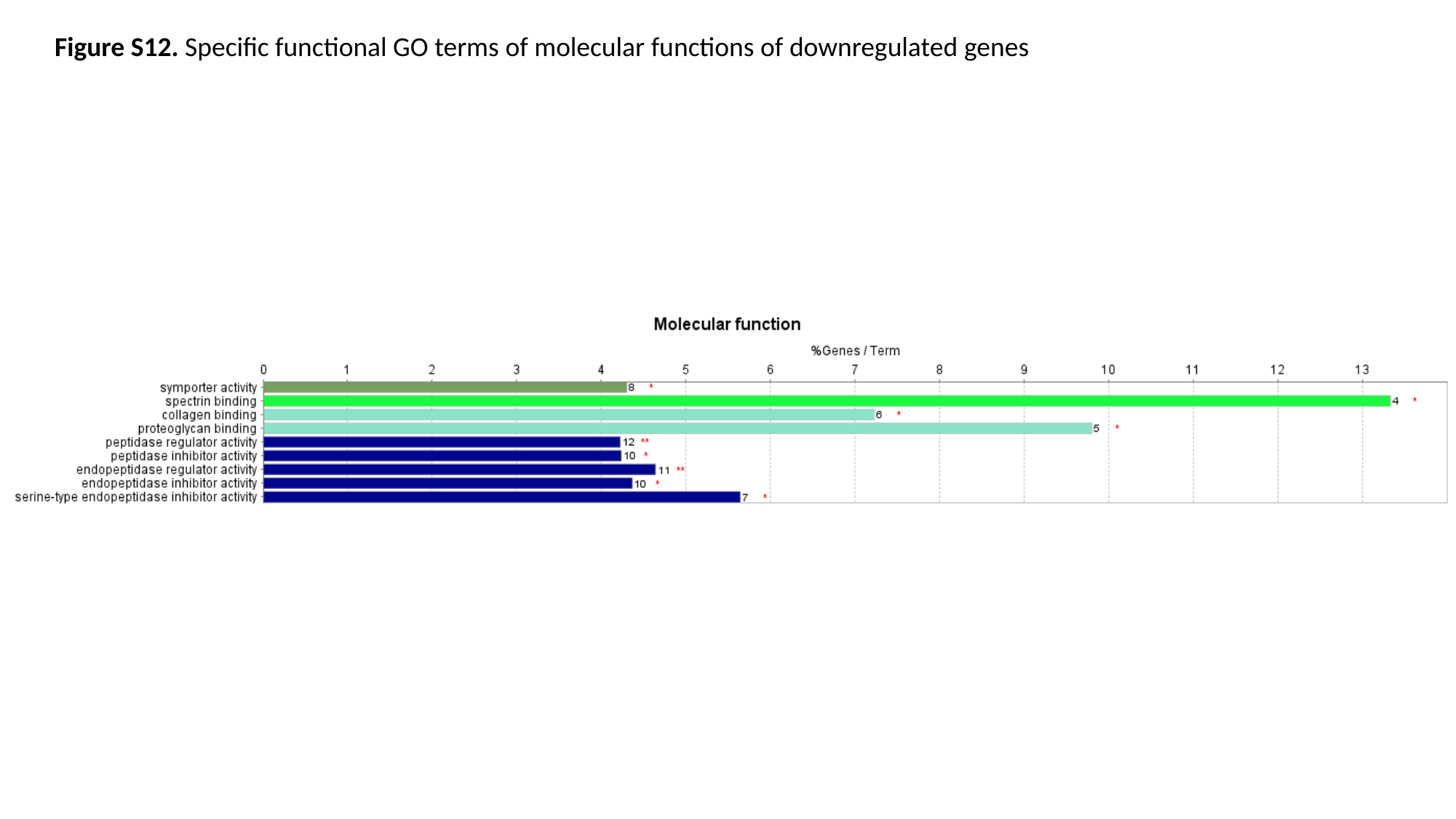

Figure S12. Specific functional GO terms of molecular functions of downregulated genes
